# Comparison of musculoskeletal networks of the primate forelimb

**DOI:** 10.1038/s41598-017-09566-7

**Published:** 2017-09-05

**Authors:** Julia Molnar, Borja Esteve-Altava, Campbell Rolian, Rui Diogo

**Affiliations:** 1 Howard University College of Medicine, Numa Adams Building, 520 W Street NW, Washington, DC 20001 USA; 2 The Royal Veterinary College, Hawkshead Lane, Hatfield, Hertfordshire, AL97TA UK; 3 University of Calgary, 3330 Hospital Drive NW, Calgary, Alberta T2N 4N1 Canada

## Abstract

Anatomical network analysis is a framework for quantitatively characterizing the topological organization of anatomical structures, thus providing a way to compare structural integration and modularity among species. Here we apply this approach to study the macroevolution of the forelimb in primates, a structure whose proportions and functions vary widely within this group. We analyzed musculoskeletal network models in 22 genera, including members of all major extant primate groups and three outgroup taxa, after an extensive literature survey and dissections. The modules of the proximal limb are largely similar among taxa, but those of the distal limb show substantial variation. Some network parameters are similar within phylogenetic groups (e.g., non-primates, strepsirrhines, New World monkeys, and hominoids). Reorganization of the modules in the hominoid hand compared to other primates may relate to functional changes such as coordination of individual digit movements, increased pronation/supination, and knuckle-walking. Surprisingly, humans are one of the few taxa we studied in which the thumb musculoskeletal structures do not form an independent anatomical module. This difference may be caused by the loss in humans of some intrinsic muscles associated with the digits or the acquisition of additional muscles that integrate the thumb more closely with surrounding structures.

## Introduction

The concept of an organism’s body as a set of semi-independent parts that maintain a level of autonomy to change (modularity) while still growing and functioning in coordinated ways (integration) continues to gain support as a central phenomenon in evolution^1–5^. A modular organization of the body allows for variation in the direction and magnitude of changes among and within parts without impairing previous functions, which promotes an organism’s capacity to generate heritable phenotypic variation^6–9^. The upper limb of primates and their close relatives is a good example of this compromise. The forelimb exhibits great diversity in anatomy, proportions, and function in primates. For example, among primates and their close relatives there are examples of forelimbs that are elongated relative to body size and used for brachiation (e.g., *Hylobates, Pongo*), attached to a membrane and used for gliding (*Cynocephalus*), and mainly freed from locomotion and specialized for manipulative activities, such as creating and using tools (*Homo*)^10, 11^. However, studies of integration and modularity in primates to date have focused exclusively on hard tissues, largely due to methodological and practical considerations (e.g., ease of measurement in skeletal collections)^5^. Therefore, although skeletal systems cannot function without actuators, such as muscles, we currently know little about how integration and modularity impact the evolution of muscles and vice-versa. To understand the evolutionary history of primates in general and the peculiar anatomy of the human lineage in particular, the musculoskeletal system must be considered in its entirety. Here, we explore Anatomical Network Analysis (AnNA) as a method to study the evolution of integration and modularity in the musculoskeletal anatomy of the primate forelimb. This approach differs from the most common studies of modularity and integration using morphometrics in its focus on the topological arrangement of parts (e.g., articulations and attachments) rather than covariation in size and shape^12, 13^. AnNA and morphometrics quantify different aspects of the organism’s morphology, thus offering complementary information about the organization and variation of morphological modules^14^. One of the main advantages of AnNA is the ability to directly compare body parts made of different tissue types, such as bones and muscles^15, 16^.

**Figure 1:**
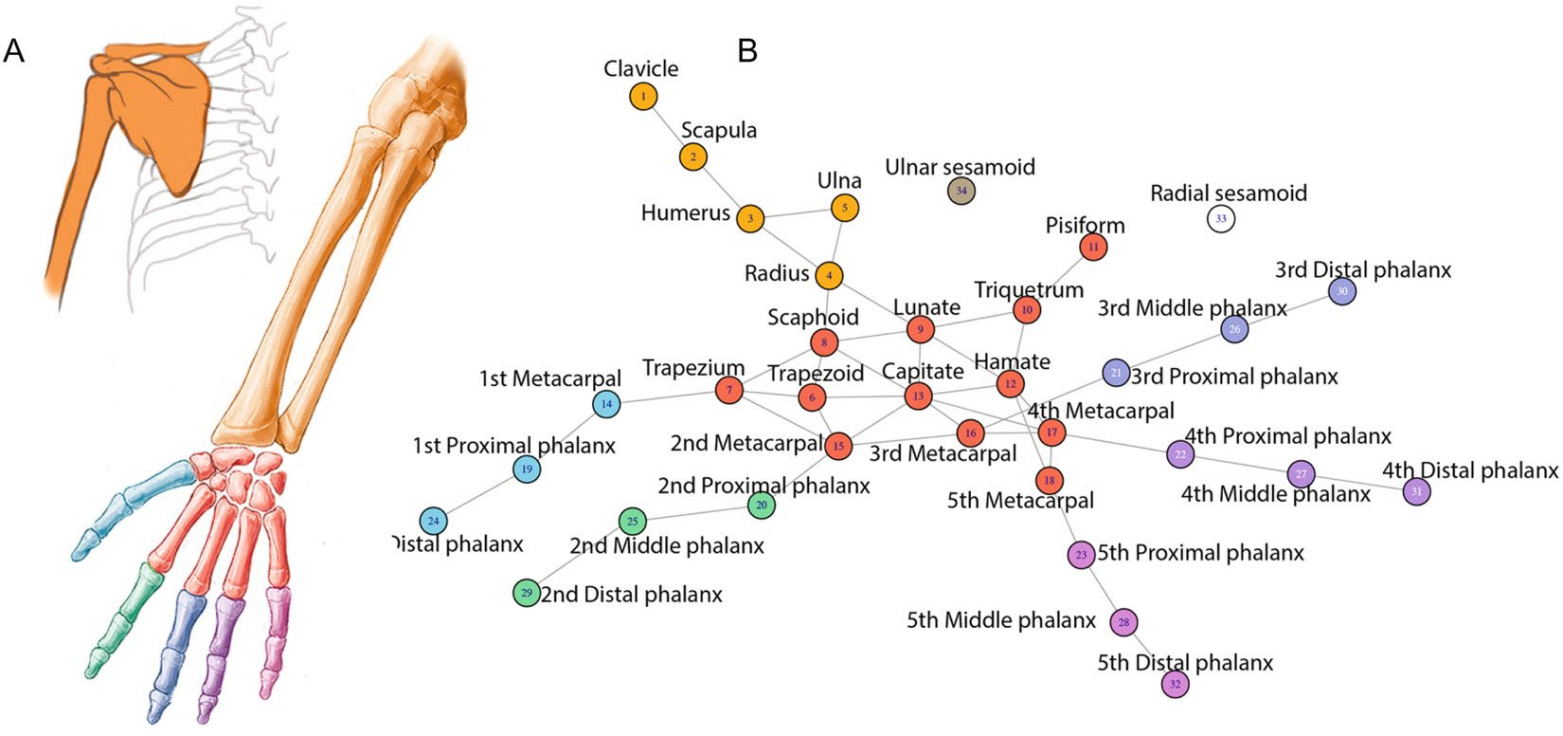
Example showing skeletal anatomy of the human forelimb (**A**) and its corresponding network model. (**B**) Every bone of the forearm is modeled in the network as a node. Links between two nodes represent the presence of a physical connection among bones. Colors indicate morphological modules. In the modeling of musculoskeletal networks the same formalization is applied, although nodes represent bones + muscles, and links represent any type of physical connection among them.

AnNA employs methods from network theory to quantify and compare the anatomy of organisms. To this end, we analyzed the musculoskeletal structures of primate forelimbs as network models (Fig. 1). These models represent the bones and muscles of the forelimb as the nodes (or vertices) of the network, and the pair-wise articulation and/or attachment between bones and muscles as the links (or edges) that connect the nodes of the network. Anatomical network models were coded as adjacency matrices in which each contact between two nodes is coded as 1 and the absence of contact between two nodes is coded as 0^12^.

We compared the overall anatomical organization of forelimbs using six network parameters (Table 1): number of anatomical structures, or nodes (N), number of physical connections, or links (K), density (D), clustering coefficient (C), path length (L), and heterogeneity (H). Nodes and links account for the number of anatomical structures modeled and the number of physical contacts among them. Density is the number of actual connections with respect to the maximum possible. Density is often used as a proxy for the complexity of a morphological structure because, in theory, more connections among parts allows more functional possibilities. Clustering coefficient is the average of the sum of connections between all neighbors of each node with respect to the maximum possible; it measures the number of triangular loops or motifs in the network. Clustering coefficient is used as a proxy for the relative amount of biological inter-dependence among nodes and of network integration. L is the average of the minimum distance between all pairs of nodes in the network. Distance is measured in number of connections, each having 1 unit length. L is used as a proxy of effective proximity (e.g., potential ability to work together) among anatomical parts. Finally, heterogeneity is the ratio between the standard deviation of connections per node and the mean number of connection per node, which provides an estimate of the irregularity of the network. Heterogeneity is used as a proxy of anisomerism; that is, how different or non-homogeneous the parts that compose the morphological structure are. Further details on the mathematical description and morphological interpretation of these parameters have been given elsewhere^13^.

**Table 1:** Summary of the morphological interpretation of network parameters (after ref. 13).

Parameter	Definition	Morphological Interpretation
Nodes (N)	Number of anatomical structures	Bones and muscles
Links (K)	Number of physical connections	Articulations and attachment
Density (D)	Ratio of the number of links with respect to the maximum number of links possible	Complexity
Clustering coefficient (C)	Number of 3-node loops or triangular motifs	Integration of parts
Path length (L)	Average of all shortest walks between every pair of nodes	Effective proximity of parts
Heterogeneity (H)	Ratio of the variance and mean of number of links per node	Anisomerism or disparity of parts

In addition to these parameters, we searched for the modular organization of forelimb networks using a community detection algorithm^17^. A network module is a group of nodes with more connections among themselves than to other nodes outside of the module. This definition agrees with the minimal definition of morphological module as a group of body parts that are more integrated among themselves than they are with other parts outside the group^5, 18^.

We explored the application of AnNA to the study of the primate forelimb using a set of hypotheses about how the network organization (parameters and modules) would vary across taxa, based on our previous works^19–25^. The theoretical framework is that different network parameters capture, or are proxies of, various features of anatomical systems, such as complexity, anisomerism, integration, and modularity (Table 1). Therefore, the first hypothesis was that taxa that share a recent common ancestor will have similar values for network parameters; i.e., the parameters will show a phylogenetic signal^26^. Because connections among anatomical structures involve developmental and functional interactions^13, 14^, our second hypothesis was that network modules represent not only topological organization but also have a developmental and functional significance. We predicted that species with uniquely derived modes of locomotion, such as ricochetal brachiation in gibbons and bipedalism in humans, and/or other functional specializations, e.g., for grasping (pincer-like morphology of *Loris* and *Nycticebus)* and enhanced manipulation (thenar musculature in *Homo*), would show unique modular architecture of the forelimb musculoskeletal system.

**Figure 2:**
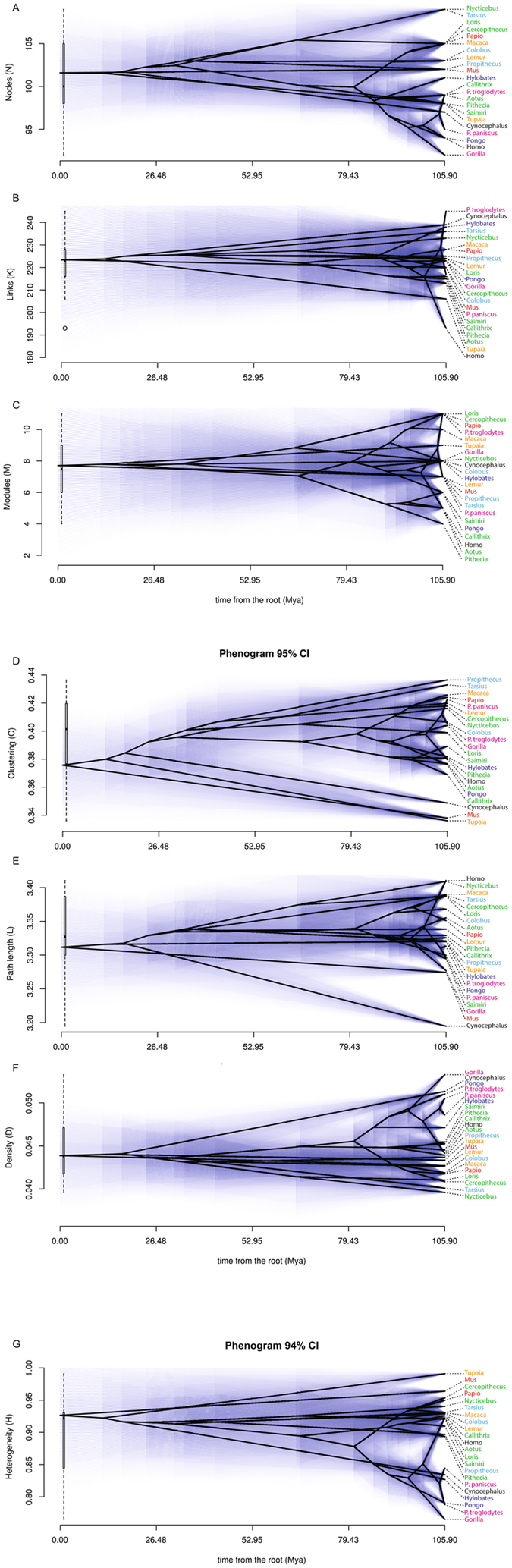
Phenograms showing the mapping of network parameters in the primate tree. The ancestral states of the phylogeny have been reconstructed using maximum likelihood (blue shades mark 95% CI). A slim box plot has been added to show the statistical distribution of values in our sample. Taxa are color-coded by locomotor type: red = mainly terrestrial; orange = semi-terrestrial; green = quadrupedal arboreal climber; blue = quadrupedal arboreal leaper; purple = brachiation; magenta = knuckle-walking; black = other. See Table 1 for definition of network parameters.

We analyzed the musculoskeletal networks of the forelimb in 22 genera representing all major extant primate clades as well as three outgroups: the primate sister-group Dermoptera (colugos), their sister-group Scandentia (tree-shrews), and their sister-group Glires (rodents and lagomorphs), represented by *Cynocephalus*, *Tupaia* and *Mus*, respectively (see Methods). Among the taxa we studied, we considered values that fall in the same interquartile range of the distribution to be similar, those that fall in different ranges to be substantially different, and those that fall in non-adjacent ranges (e.g., below the 1^st^ quartile versus above the 2^nd^ quartile) to be very different. Box plots showing the distribution of the data are included in each phenogram (Fig. 2).

## Results

### Parameters

The architecture of the forelimb, as captured by network musculoskeletal parameters, is specific to each taxon (Table 2), but shows a limited range of variation within primates (Fig. 2). We found little evidence of similarity among taxa based on their phylogenetic proximity; most network parameters lack a phylogenetic signal (Table S1). Only nodes and clustering coefficient show a statistically significant phylogenetic signal, with Pagel’s λ and Blomberg’s *k* estimates differing in the relative strength of the signal (λ and *k* tend toward 1 when traits vary under a Brownian Motion model in the tree). Nodes shows a strong signal in Pagel’s test (λ = 0.92, p-value = 0.009) but a mild signal in Blomberg’s test (*k* = 0.45, p-value = 0.013), while clustering coefficient shows a relatively strong signal in both tests (λ = 0.92, p-value = 0.004; *k* = 0.73, p-value = 0.001). This means that the taxa that are most closely related have similar values for the number of elements and their integration, but this similarity does not seem to translate to other topological features. The skeletal component of the forelimb was studied separately (Tables S2 and S4), but the number and arrangement of bones does not vary enough among taxa for a meaningful comparative analysis.

**Table 2:** Values of network parameters measured in forelimb musculoskeletal networks. N, nodes; K, links; D, density; C, clustering coefficient; L, path length; H, heterogeneity; and M, number of connectivity modules.

**Taxa**	**N**	**K**	**D**	**C**	**L**	**H**	**M**
*Mus musculus*	102	220	0.043	0.338	3.274	0.964	7
*Tupaia sp*.	98	206	0.043	0.336	3.320	0.991	9
*Cynocephalus volans*	97	239	0.051	0.349	3.195	0.834	8
*Lemur catta*	103	224	0.043	0.420	3.338	0.928	7
*Propithecus verreauxi*	102	225	0.044	0.436	3.321	0.897	7
*Loris tardigradus*	105	224	0.041	0.399	3.387	0.921	11
*Nycticebus pygmaeus*	109	233	0.040	0.416	3.409	0.940	8
*Tarsius syrichta*	109	236	0.040	0.433	3.389	0.931	7
*Callithrix jacchus*	99	216	0.045	0.369	3.326	0.926	5
*Saimiri sciureus*	98	216	0.045	0.389	3.295	0.910	6
*Aotus nancymaae*	99	213	0.044	0.377	3.355	0.922	5
*Pithecia pithecia*	98	215	0.045	0.381	3.329	0.894	4
*Colobus guereza*	103	220	0.042	0.406	3.368	0.930	8
*Cercopithecus diana*	105	223	0.041	0.418	3.387	0.953	11
*Papio anubis*	105	228	0.042	0.424	3.351	0.950	11
*Macaca fascicularis*	105	228	0.042	0.426	3.390	0.930	10
*Hylobates lar*	101	238	0.047	0.382	3.320	0.827	8
*Pongo pygmaeus*	94	223	0.051	0.376	3.300	0.790	6
*Gorilla gorilla*	92	223	0.053	0.404	3.277	0.765	8
*Pan troglodytes*	99	245	0.051	0.405	3.313	0.788	11
*Pan paniscus*	95	217	0.049	0.423	3.296	0.845	7
*Homo sapiens*	94	193	0.044	0.380	3.410	0.923	5

Despite the lack of a statistically significant phylogenetic signal, some variation in network parameters is evident among clades from the distribution of values in the sample. Non-primate taxa have relatively low clustering coefficients and path lengths (below the 1^st^ quartile and 2^nd^ quartiles, respectively) **(**Fig. 2 **)**. The dermopteran *Cynocephalus* has the highest density of any non-hominoid taxon analyzed (N.B. hominoids include apes and humans). In strepsirrhine primates, nodes is above the 3^rd^ quartile. New world monkeys (NWM, or platyrrhines) have densities above the 2^nd^ quartile and nodes and clustering coefficients below the second quartile. In contrast, OWM all have nodes between the 2^nd^ and 3^rd^ quartiles, densities below the 2^nd^ quartile, and clustering coefficients and heterogeneity above the 2^nd^ quartile. Hominoids have nodes below the 2^nd^ quartile (except *Hylobates)* but densities above the 3^rd^ quartile; excluding *Homo* (which is average on both counts), they have path lengths below the 2^nd^ quartile and heterogeneity below the 1^st^ quartile.

### Modularity

The number of musculoskeletal network modules in the forelimb ranges from four to 11 (Table S3) without a significant phylogenetic signal (λ = 0.411, p-value = 1; *k* = 0.136, p-value = 0.742). The number of modules in NWM falls below the 1^st^ quartile, whereas in OWM it falls above the 2^nd^ quartile (Fig. 2C). Notably, the number of modules varies greatly between the closely related *Pan troglodytes* (common chimps), *P. paniscus* (bonobos), and *H. sapiens*: common chimps share with some OWM and *Loris* that highest number of modules (11), while humans have five, one of the lowest numbers among primates (below 1^st^ quartile). Bonobos fall slightly below the 2^nd^ quartile, with seven modules. Common chimps are reasonably similar to bonobos in terms of locomotion and manual dexterity^27^, so there is no immediately obvious functional reason for this dramatic difference in number of modules.

**Figure 3:**
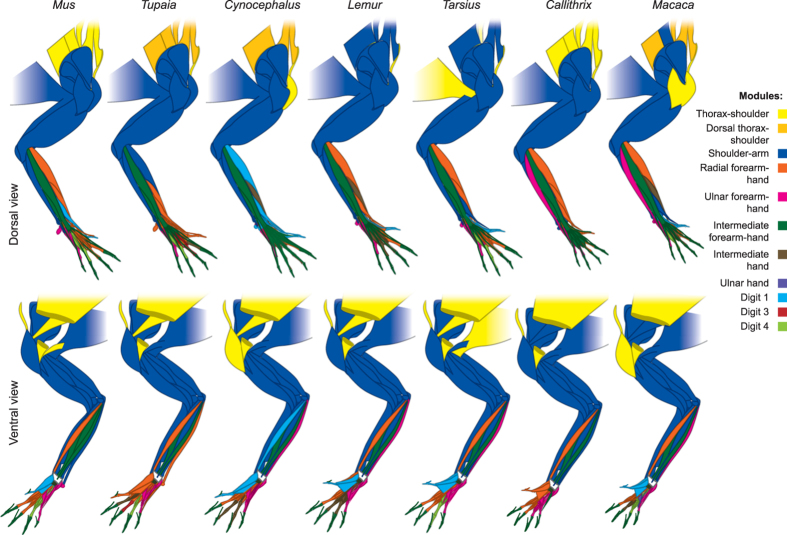
Illustration showing connectivity modules identified in the forelimb muscles and bones of representative members of the non-hominoid taxa analyzed for the present work, as listed in Table S5. Colors indicate modules that encompass similar anatomical regions among taxa, as listed at the right. To conserve space, only a single representative each of Strepsirrhini, OWMs, and NWMs is illustrated.

**Figure 4:**
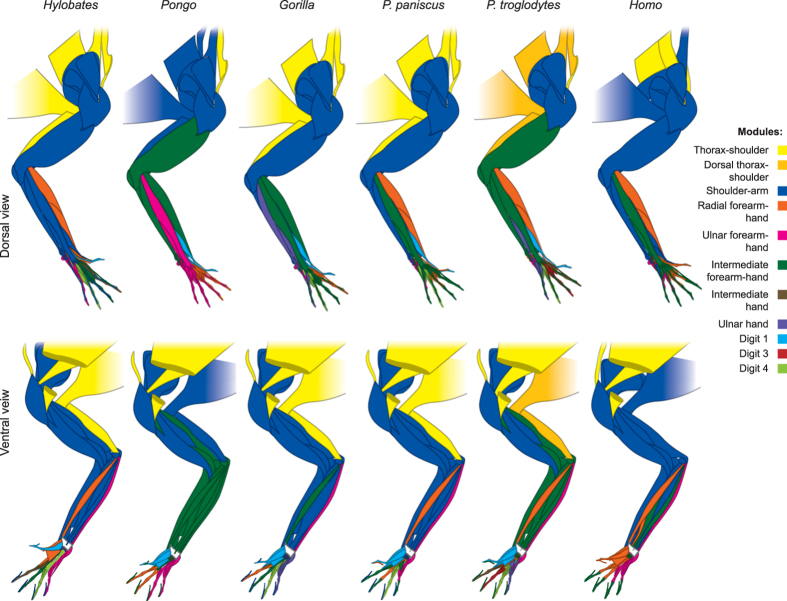
Illustration showing connectivity modules identified in the forelimb muscles and bones of representative members of Hominoidea, as listed in Table S5. Colors indicate modules that encompass similar anatomical regions among taxa, as listed on the right.

All taxa have the following five modules, with minor modifications in each taxon: thorax-shoulder, shoulder-arm, and radial, intermediate, and ulnar forearm-hand (Figs 3–4; Tables S4–5). The thorax-shoulder module (yellow) mainly consists of bones of the axial skeleton and muscles that anchor the forelimb to the trunk. This module is sometimes subdivided into dorsal (orange: occipital, vertebrae, rhomboideus muscles, levator scapulae and claviculae - where present - and latissimus dorsi) and ventral (yellow: ribs, sternum, clavicle, serratus anterior, subclavius, pectoralis major and minor) components. Deltoideus and dorsoepitrochlearis are sometimes included in the shoulder module and sometimes in the scapula + arm module. The shoulder-arm module (blue) includes muscles that originate from the girdle and insert on the arm, as well as some forearm muscles. Most commonly, it consists of the scapula, humerus, radius, and ulna in addition to the muscles originating on the girdle or latissimus dorsi and inserting on the humerus (supraspinatus, infraspinatus, teres minor, subscapularis, deltoideus, coracobrachialis, panniculus carnosus) or radius and/or ulna (biceps brachii, triceps, dorsoepitrochlearis, epitrochleoanconeus, anconeus), and the brachialis. The forearm muscles most commonly included in this module are forearm supinators/pronators and wrist flexors (pronator teres and quadratus, brachioradialis, supinator, palmaris longus). The flexor digitorum superficialis, extensor carpi ulnaris, and abductor pollicis longus are also included in this module in many of the taxa we studied.

The distal modules of the forearm and hand are more variable than the more proximal thorax-shoulder and shoulder-arm modules, often being subdivided or including different muscles in different taxa. That is, anatomical regions that directly contact the external environment (e.g., food items; substrate during locomotion) are more variable not only morphologically but also in terms of their deep network organization. Most commonly, there are two radial modules: one consisting of forearm muscles, radial carpals, and digits 2–3 (orange) and the other consisting of digit 1 and muscles inserting on digit 1 (turquoise). In several cases, there is no distinct digit 1 musculoskeletal module, despite the existence of a distinct bone-only module for this digit (including *Homo*, as discussed below). The intermediate forearm-hand module (dark green) usually consists of the forearm muscles that attach to digits 2–5 (extensor digitorum, extensor indicis, extensor digiti minimi, flexor digitorum superficialis and profundus) and, often, the middle and distal phalanges of digits 2–5. Other times, a separate intermediate hand module (brown) and/or digit 3 module (red) is present. In hominoids, the precise phalanges included in the intermediate forearm-hand module are highly variable. Some or all of the lumbricales, intermetacarpales, interossei, and/or flexores breves profundi are included in this module as well. In *Hylobates*, the forearm muscles usually included in this module are included in the shoulder-arm module instead, whereas in *Pongo* and *P. troglodytes* this module includes most forearm and some arm muscles. The ulnar forearm-hand module (pink) is often divided into digit 4 (light green), ulnar hand (purple), and ulnar forearm (pink). In several taxa (e.g., *P. troglodytes*) there are individual modules comprising each of the five digits.

## Discussion

### What does AnNA tell us about the anatomy of primate forelimbs?

The phylogenetic context of primate taxa is sufficient to explain the variation in the number of bones and muscles in their forelimbs. However, despite this constraint in the number of parts, the way in which these parts are arranged in the limb, which creates differences in complexity, modularity, and anisomerism, is independent of primate phylogenetic history. The only exception is the evolution of the overall integration (as measured by clustering) which shows a stronger phylogenetic signal than the number of parts. This result provides evidence that, although the number of bones and muscles of the forelimb is indeed dependent on the evolutionary history of primates, the way these parts are organized or arranged varies independently, presumably adapting to the specific behavioral and locomotor needs of each taxon. Because no comparable data (i.e., musculoskeletal limb network data) are available for other tetrapods, it is difficult to assess the biological significance of differences in parameters between taxa.

Only anisomerism (measured with the proxy H, heterogeneity) seems to be more directly related to function **(**Fig. 2G **)**. Among primates, hominoids have the lowest heterogeneity (mean heterogeneity in hominoids: 0.823, in non-hominoid primates: 0.923; a difference larger than one standard deviation). Most taxa in the analysis are primarily arboreal quadrupeds (e.g., *Loris, Propithecus, Callithrix, Saimiri*), running along branches and leaping among trees. The taxa that fall above the 3^rd^ quartile include the rodent *Mus*, the tree-shrew *Tupaia*, and the OWM *Papio*, which are primarily terrestrial quadrupeds (the vast majority of *Tupaia* species are terrestrial^28^). Those below the 1^st^ quartile include apes and the dermopteran *Cynocephalus*, which uses its patagium to glide. Asian apes typically engage in below-branch suspensory behaviors when moving in an arboreal environment (e.g., ricochetal brachiation in gibbons^29^). African apes divide their time between arboreal and terrestrial locomotion. In terrestrial settings, which makes up between 30% (bonobos) and 95% (mountain gorillas) of time spent in locomotion^30^, African apes frequently use a specialized form of locomotion known as knuckle-walking, which is itself associated with derived skeletal traits in the wrist^31^. In arboreal environments, African apes are most often observed climbing vertically and clambering between supports using all limbs, but brachiate infrequently. Several derived musculoskeletal features of the limbs in apes may contribute to vertical climbing and suspensory behavior, including long forelimbs relative to hind limbs, a torso shape and scapular position that facilitates adduction of the shoulder, a reduced ulnar styloid with increases the range of adduction at the carpus, and generally larger flexor musculature (reviewed in Ward^31^).

The apparent relationship between anisomerism and function is not perfect, however: the OWM *Cercopithecus* has a high value for anisomerism despite being an arboreal quadruped, and *Homo* has an average value despite being a terrestrial biped. We can only speculate that this anisomerism is related to division of labor among muscles: the more connected muscles coordinate generic motor functions by attaching to many bones at the same time (e.g., the flexor digitorum superficialis flexes all digits and helps to flex the wrist), while the muscles connected to fewer bones have specialized local, fine-tuning functions (e.g., the abductor digiti minimi abducts the 5th digit). Thus, taxa requiring more complex coordination of generic and local motor functions, such as arboreal quadrupeds navigating in a complex three-dimensional environment with substrates of varying size and distance, might benefit from having higher heterogeneity and division of labor among the connections of their muscles. The lower heterogeneity among non-human hominoids would in turn indicate that suspensory behaviors, and knuckle-walking in African apes, do not require extensive specialization of forelimb musculoskeletal connections and nodes, even though many features of the ape *skeleton* appear adaptive for both knuckle-walking and vertical climbing. In this context, the higher heterogeneity of humans among hominoids might reflect further compartmentalization of its forelimb for both gross and fine manipulative activities.

Surprisingly, the forelimb network parameters of the two most closely related taxa in our analysis, common chimps (*Pan troglodytes*) and bonobos (*Pan paniscus*), differ markedly (e.g., links, modules; Fig. 2). This example shows how small changes in the presence/attachments of muscles can dramatically change the network organization of the system as a whole without profoundly affecting locomotion and other functions of the forelimb, as bonobos and common chimps are similar in these respects.

### What does AnNA tell us about modular evolution of the primate forelimb?

In general, the modules of the proximal limb are similar between the outgroup taxa and primates, but those of the distal limb show substantial variation **(**Figs 3–4). In the sister-group of all other primates, the strepsirrhines, the dorsal shoulder module (orange) is part of the larger shoulder-arm module (blue). In addition, unlike the case in the dermopteran *Cynocephalus* and the rodent *Mus*, not all bones of digit 1 in *Lemur* are part of the ‘digit 1’ module (turquoise). The main difference between *Lemur* and *Propithecus* and all the other taxa analyzed for this study is that the structures of the intermediate hand module (brown) of these two genera are integrated into other modules in other primates; for example, into the larger radial forearm and hand module (orange) in many taxa and into the digit 4 module in *Tarsius*. In this case, the difference appears to reflect an autapomorphy of the Lemuroidea, as among the taxa we studied only *Lemur* and *Propithecus* have such an intermediate hand module including parts of both digits 3 and 4 and associated muscles. The most distant outgroup taxon *Mus* has well-defined musculoskeletal modules related to movements of digits 1, 4, and 5 (turquoise, light green, and pink, respectively), but not digits 2 and 3. In the sister-group of all other primates, the strepsirrhines, the dorsal shoulder module (orange) is part of the larger shoulder-arm module (blue). In addition, unlike the case in the dermopteran *Cynocephalus* and the rodent *Mus*, not all bones of digit 1 in *Lemur* are part of the ‘digit 1’ module (turquoise). Interestingly, *Cynocephalus* is generally more similar to phylogenetically basal primates than is the tree-shrew *Tupaia* in terms of the composition of individual modules. This similarity is notable because only a few anatomical features have been proposed so far to support the Dermoptera-Primates sister-group relationship that was put forward by genetic studies^32, 33^. For instance, the radial forearm and hand module of *Tupaia* has many more components (including, e.g., metacarpals 2 and 3) than those of *Cynocephalus* and strepsirrhines such as *Lemur*, and *Propithecus*.

The number of musculoskeletal modules in *Colobus* (eight; Table 2) probably represents the plesiomorphic condition in anthropoids (platyrrhines + catarrhines). As other hominoids have eight (*Gorilla, Hylobates*) and six (*Pongo*) modules, and *Colobus -* a member of the sister-group of all other OWM, and the taxon thought to have the most plesiomorphic muscle anatomy among the OWM in this study^32, 33^ - also has eight modules, the presence of eight modules is most parsimoniously interpreted as plesiomorphic for hominoids, and for anthropoids in general. Therefore, compared with this number of modules, common chimps show decreased musculoskeletal integration (11 modules) and humans show increased musculoskeletal integration (only 5 modules). *Colobus* has an intermediate forearm module (dark green) that is very similar to that of NWM in that it includes the proximal and distal phalanges of digits 2, 3, 4 and 5 and the distal phalanx of digit 1 (digit 1 is variably reduced in size in *Colobus* specimens, and is completely missing in some of them), plus five separate modules including the muscles and bones of each individual digit (except digit 2, as the metacarpal and proximal phalanx of this digit are included in the digit 1 module, shown in turquoise). Thus, the plesiomorphic forelimb organization of anthropoids has more modules related to movement of individual digits compared to that of NWM, which also have fewer modules than most other primates. The opposite is seen in the Cercopithecinae (*Macaca*, *Papio*, and *Cercopithecus*), in which the bones of the digits may be divided into as many as to six separate modules, contributing to the greater overall number of modules in this OWM subfamily. In this case, the difference may be directly related to function because OWM generally have a greater ability to coordinate individual digits than NWM^34–36^.

### What does AnNA tell us about the evolution of digits?

We predicted that humans would have separate modules including the musculoskeletal structures of the thumb because humans rely heavily on manipulative abilities and activities, and because they have more muscles related to the movement of the thumb than any other primates^19, 37^. Therefore, it is surprising that humans are one of the few primates we studied that lack a distinct digit 1 bone-muscle module. This module, comprising both phalanges of the thumb (with or without the trapezium and metacarpal 1) and at least 70% of the muscles that attach to these bones (where present: adductor pollicis, flexor brevis profundus 2, opponens pollicis, flexor pollicis longus and brevis, abductor pollicis longus and brevis, extensor pollicis longus and brevis) but no other structures, was present in the other four hominoids, the OWM *Cercopithecus* and *Papio*, the four strepsirrhines, and *Tarsius* **(**Figs 3–4 **)**.

The lack of an individual thumb module in humans may be explained by the loss of other intrinsic hand muscles. While the number of thumb muscles has increased during human evolution, many of the serial hand muscles (e.g., contrahentes) that are related to the movement of each of the other four digits have been lost^37^. Therefore, the presence of more serial muscles inserting on digits 2–4 may result in at least some of them being included in their own modules, and therefore as a by-product – i.e., merely by exclusion - digit 1 is also a separate module in many non-human primates, contrary to the pattern observed in humans. Alternatively or in combination, from a functional perspective, the coalition of the thenar plus radial group in *Homo* vs. hypothenar and ulnar groups in other hominoids could be related to an increased demand for forceful precision grips in humans; that is, recruiting the radial carpal muscles to help stabilize the thenar group that is thought to have played an important role in the evolution of manual dexterity in humans^38–40^. In other hominoids, the thumb/radial group and hypothenar/ulnar group are not as clearly separated as they are in *Homo* (e.g., the other hominoids appear to have a separate thenar group not part of the antebrachium: Fig. 4: turquoise).

We also predicted that *Loris* and *Nycticebus* would share unique modules related to the digits because of the unusual configuration of their hands in which digit 2 is reduced and digit 1 is widely separated from the other digits. However, the digit modules in these two taxa are similar to those of other strepsirrhines: the middle and distal phalanges are part of the intermediate forearm module, and the proximal phalanges, metacarpals, and intrinsic hand muscles comprise a thumb module, ulnar and radial forearm-hand modules, and an intermediate hand module including extensor carpi radialis longus or brevis. In *Nycticebus* there is a separate digit 4 module rather than an intermediate hand module, and the ulnar forearm-hand module includes only hand muscles. In *Loris* there are separate digit 2, 3 and 4 modules rather than a single intermediate hand module.

### Limitations and comparison with morphometric studies on integration

In defining nodes and links, we chose to include only bones and muscles - not ligaments - to make our results comparable to previous studies e.g.^16, 41^. Because of this methodological choice, our networks do not include connections such as the ulnar collateral ligament between the ulna and triquetrum and pisiform in hominoids, which plays an important role in wrist function. In the future, we plan to expand our anatomical network studies to include other soft tissues such as ligaments, blood vessels, and nerves.

Most studies of morphological integration and modularity in the primate forelimb to date have focused on the analysis of co-variation patterns in the proportions (size and shape) of entire limbs or of specific bones (reviewed in ref. 5). The results of AnNA presented here are based, instead, on the analysis of the topological arrangement of bone and muscle connections. Thus, inferences of modularity by AnNA are complementary to those derived from morphometrics studies^14^. The precise causal relationship between proportions and connections is still unknown (for a broader discussion see^13, 14, 42^); however, the presence of this relationship has been demonstrated for the human skull, where connectivity modules capture skeletal units of growth as measured by shape changes^42^. Because AnNA is better suited to compare disparate anatomies, its results can recover some of major anatomical differences among groups, such as the difference between hominines (gorillas, chimpanzees and humans) and other primates in the organization of the carpus; however, it does not provide a finer-grained view of the differences between closely related taxa where variability in the pattern of connections is reduced, and most disparity arises due to changes in size and shape. Hence, AnNA offers an independent perspective on biological form, focused on the way in which constituent anatomical parts are interrelated or arranged, and complementary to changes in size and shape. We hope the present work will pave the way for future studies on the musculoskeletal anatomy of specific primate subgroups and on comparisons with other mammals and tetrapods, encouraging researchers to combine AnNA and morphometrics to study morphological modularity. Such an integrative approach will provide a more comprehensive understanding of complexity and evolvability of the limbs within primates and among tetrapods in general.

### Summary

Anatomical network analysis describes the organization of topological connections between anatomical structures, yielding quantitative parameters that can be used as proxies for anatomical features such as complexity and integration. A major advantage of this method is the ability to compare parameters across taxa to test hypotheses such as whether they to be more similar among taxa that share a recent common ancestor or that share locomotor or other functional characteristics. In the current study, we used anatomical network analysis to obtain novel information about evolutionary changes in complexity, anisomerism, modularity, and integration in the upper limb of primates.

## Methods

### Specimens and data collection

We included in our analysis members of all major extant primate groups, as well as of the three living groups more closely related to Primates: Glires (including rodents such as *Mus*), Dermoptera (or colugos, represented by *Cynocephalus*) and Scandentia (or tree shrews, represented by *Tupaia*). The musculoskeletal data for primates and these three taxa was taken from dataset provided by Diogo and Wood^20^, which was based on their own dissections and an extensive review of the literature; multiple specimens were represented in this dataset, and the most common configuration reported for each taxon was coded in the network matrices. Additional data relating to skeletal articulations were compiled from observations of skeletal specimens housed at the Harvard University Museum of Comparative Zoology and the American Museum of Natural History and from the literature^11, 43–48^.

### Anatomical network analysis

**Figure 5:**
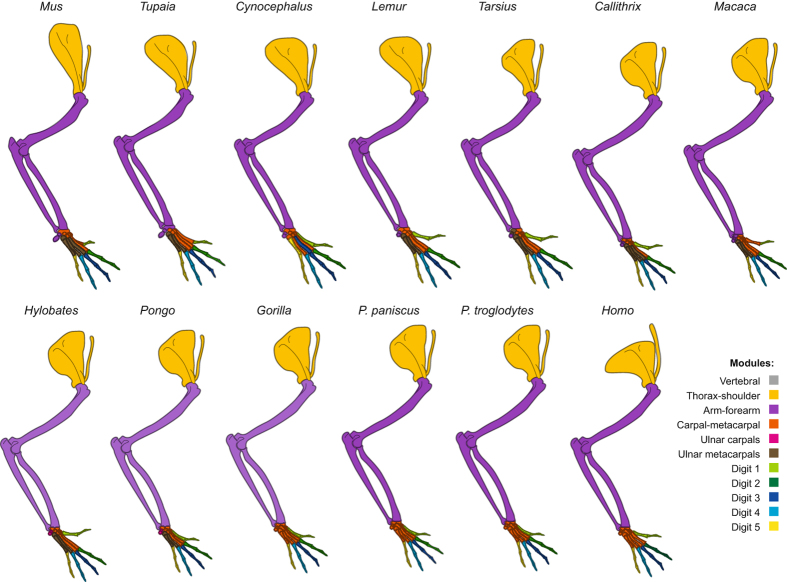
Illustration showing connectivity modules identified in the forelimb bones of representative members of the taxa analyzed for the present work, as listed in Table S4. Colors indicate modules that encompass similar anatomical regions among taxa, as listed at the right. To conserve space, only a single representative each of Strepsirrhini, OWM, and NWM is illustrated.

We built unweighted, undirected network models of the musculoskeletal anatomy of the forelimb of 19 primate taxa and the 3 out-groups. Anatomical networks were coded as adjacency matrices in Excel sheets and analyzed in R^49^ using the package *igraph* ^50^. In defining nodes and links (see Fig. 1), we chose to include only bones and muscles - not ligaments - to make our results comparable to previous studies^15^. Because of this methodological choice, our networks do not include connections such as the ulnar collateral ligament between the ulna and triquetrum and pisiform in hominoids, which plays an important role in wrist function. For completeness, we also analyzed the skeletal components separately and created skeletal networks to this end (Fig. 5). Skeletal networks include only bones (as the nodes of the network) connected by their articulations (as the links). We offer an extended discussion of results in Supplementary Materials.

For every network, parameters were measured using functions implemented in the package *igraph*. We delimited the modules of the anatomical networks using a standard random walk algorithm, using the function cluster_walktrap of *igraph*. The heuristics of this algorithm is that short random walks (we used random walks of 3 steps) tend to concatenate nodes within the same module^51^. This allows us to find groups of nodes (modules) more densely connected among themselves than to nodes outside of the module. The quality of the identified partitions is evaluated using the optimization function modularity Q defined by Newman and Girvan^52^, which is commonly used to assess whether the partition identified by a community detection algorithm is better that what is expected at random. Q will be close to 0 if the number of links within modules is no better than that expected at random; Q will be closer to 1 if the modules identified deviate from what is expected for a random network. According to Newman and Girvan’s observations, usual values of strongly modular networks range between 0.3 and 0.7. The expected error of Q was calculated using a jackknife procedure, where every link is an independent observation. Additionally, we performed a two-sample Wilcoxon rank-sum test on the internal vs. external connections of every module, in order to estimate their statistical significance. According to the general definition of a module as a group of nodes highly connected among themselves and poorly connected to nodes in other groups, we expect internal connections to be significantly higher than external connections (H_0_: *K*_*internal*_ = *K*_*externa*l_; H_A_: *K*_*internal*_ > *K*_*external*_). Lower p-values tell us to reject H_0_, and hence we can assume the alternative hypothesis that the nodes of the module are more connected among themselves than to other nodes outside the module, is supported. In other words, the module identified is not expected by a random grouping of nodes.

### Phylogenetic analysis

We tested the assumption of phylogenetic independence of network parameters nodes, links, density, clustering, heterogeneity, and of the number of modules of forelimb networks using a calibrated phylogeny of the 22 taxa included in this study^53, 54^. We performed a Pagel’s test and a Blomberg’s test using the function phylosig of the package *phytools* ^55^ in R. For both indexes, a value close to zero indicates phylogenetic independence and a value of one indicates that species’ network parameters are distributed as expected under Brownian Motion (i.e., there is phylogenetic signal)^26^. If p < 0.05 we reject H_0_ and we cannot rule out the presence of a phylogenetic signal in that network parameter. To show visually the evolution of network parameters in primates we used the function phenogram in *phytools*. This function maps parameters on the consensus calibrated phylogeny and estimate the values of internal nodes using maximum likelihood, interpolating the states along each branch as in Felsenstein^56^ and calculating 95% confidence intervals.

## Electronic supplementary material

Tables S1-S5

Dataset 1

## References

[1] Wagner GP, Pavlicev M, Cheverud JM (2007). The road to modularity. Nat. Rev. Genet..

[2] Müller GB (2007). Evo–devo: extending the evolutionary synthesis. Nat. Rev. Genet..

[3] Klingenberg CP (2008). Morphological integration and developmental modularity. Annu. Rev. Ecol. Evol. Syst..

[4] Goswami A, Binder WJ, Meachen J, O’Keefe FR (2015). The fossil record of phenotypic integration and modularity: A deep-time perspective on developmental and evolutionary dynamics. Proc. Natl. Acad. Sci..

[5] Esteve-Altava, B. In search of morphological modules: a systematic review. *Biol. Rev*. **92**, 1332–1347 (2016). 10.1111/brv.12284 27245113

[6] Kirschner M, Gerhart J (1998). Evolvability. Proc. Natl. Acad. Sci..

[7] Raff EC, Raff RA (2000). Dissociability, modularity, evolvability. Evol. Dev..

[8] Rolian C (2009). Integration and evolvability in primate hands and feet. Evol. Biol..

[9] Pavlicev M, Hansen TF (2011). Genotype-Phenotype Maps Maximizing Evolvability: Modularity Revisited. Evol. Biol..

[10] Napier JR (1961). Prehensility and opposability in the hands of primates. Symp Zool Soc Lond.

[11] Panyutina, A. A., Korzun, L. P. & Kuznetsov, A. N. Forelimb Morphology of Colugos. In *Flight of Mammals: From Terrestrial Limbs to Wings* 51–114 (Springer International Publishing, 2015).

[12] Esteve-Altava B, Marugán-Lobón J, Botella H, Rasskin-Gutman D (2011). Network models in anatomical systems. J. Anthropol. Sci..

[13] Rasskin-Gutman D, Esteve-Altava B (2014). Connecting the dots: anatomical network analysis in morphological EvoDevo. Biol. Theory.

[14] Esteve-Altava, B. Challenges in identifying and interpreting organizational modules in morphology. *J. Morphol*. **278**, 960–974 (2017). 10.1002/jmor.20690 28466514

[15] Diogo R, Esteve-Altava B, Smith C, Boughner JC, Rasskin-Gutman D (2015). Anatomical network comparison of human upper and lower, newborn and adult, and normal and abnormal limbs, with notes on development, pathology and limb serial homology vs. homoplasy. PLOS ONE.

[16] Esteve-Altava B, Diogo R, Smith C, Boughner JC, Rasskin-Gutman D (2015). Anatomical networks reveal the musculoskeletal modularity of the human head. Sci. Rep..

[17] Fortunato S (2010). Community detection in graphs. Phys. Rep..

[18] Eble, G. J. Morphological modularity and macroevolution. in *Modularity: understanding the development and evolution of natural complex systems* (eds. Callebaut, W. & Rasskin-Gutman, D.) 221–238 (The MIT Press, 2005).

[19] Diogo, R. & Abdala, V. *Muscles of Vertebrates–comparative anatomy, evolution, homologies and development*. (Science Publishers, 2010).

[20] Diogo, R. *et al*. Photographic and descriptive musculoskeletal atlas of gorilla: with notes on the attachments, variations, innervation, synonymy and weight of the muscles. (CRC Press, 2010).

[21] Diogo, R. & Wood, B. *Comparative anatomy and phylogeny of primate muscles and human evolution*. (Taylor and Francis, 2012).

[22] Diogo, R. *et al*. Photographic and Descriptive Musculoskeletal Atlas of Gibbons and Siamangs (Hylobates): With Notes on the Attachments, Variations, Innervation, Synonymy and Weight of the Muscles. (CRC Press, 2012).

[23] Diogo, R., Potau, J. M. & Pastor, J. F. *Photographic and descriptive musculoskeletal atlas of chimpanzees (*Pan*) - with notes on the attachments, variations, innervation, synonymy and weight of the muscles*. (Taylor and Francis, 2013).

[24] Diogo, R. *et al*. Photographic and descriptive musculoskeletal atlas of orangutans: with notes on the attachments, variations, innervations, function and synonymy and weight of the muscles. (CRC Press, 2013).

[25] Diogo, R. *et al*. Photographic and descriptive musculoskeletal atlas of bonobos - with notes on the weight, attachments, variations, and innervation of the muscles and comparisons with common chimpanzees and humans. (Springer, 2017).

[26] Münkemüller T (2012). How to measure and test phylogenetic signal. Methods Ecol. Evol..

[27] Neufuss J, Humle T, Cremaschi A, Kivell TL (2017). Nut-cracking behaviour in wild-born, rehabilitated bonobos *(Pan paniscus):* a comprehensive study of hand-preference, hand grips and efficiency. Am. J. Primatol..

[28] Emmons, L. H. *Tupai: a field study of Bornean treeshrews*. **2**, (Univ of California Press, 2000).

[29] Usherwood JR, Larson SG, Bertram JE (2003). Mechanisms of force and power production in unsteady ricochetal brachiation. Am. J. Phys. Anthropol..

[30] Crompton RH, Sellers WI, Thorpe SK (2010). Arboreality, terrestriality and bipedalism. Philos. Trans. R. Soc. B Biol. Sci..

[31] Ward, C. V. Postcranial and locomotor adaptations of hominoids. *Handb. Paleoanthropology* 1363–1386 (2015).

[32] Diogo R, Wood B (2011). Soft-tissue anatomy of the primates: phylogenetic analyses based on the muscles of the head, neck, pectoral region and upper limb, with notes on the evolution of these muscles. J. Anat..

[33] Diogo, R. & Wood, B. A. *Comparative anatomy and phylogeny of primate muscles and human evolution*. (Science Publishers (CRC Press), 2012.

[34] Fragaszy DM (1983). Preliminary quantitative studies of prehension in squirrel monkeys *(Saimiri sciureus)*. Brain. Behav. Evol..

[35] Schieber MH (1991). Individuated finger movements of rhesus monkeys: a means of quantifying the independence of the digits. J. Neurophysiol..

[36] Christel MI, Fragaszy D (2000). Manual function in *Cebus apella*. Digital mobility, preshaping, and endurance in repetitive grasping. Int. J. Primatol..

[37] Diogo R, Ziermann JM, Linde-Medina M (2015). Specialize or risk disappearance – empirical evidence of anisomerism based on comparative and developmental studies of gnathostome head and limb musculature. Biol. Rev..

[38] Marzke MW (1997). Precision grips, hand morphology, and tools. Am. J. Phys. Anthropol..

[39] Rolian C, Lieberman DE, Zermeno JP (2011). Hand biomechanics during simulated stone tool use. J. Hum. Evol..

[40] Marzke MW (2013). Tool making, hand morphology and fossil hominins. Philos. Trans. R. Soc. B Biol. Sci..

[41] Diogo R, Esteve-Altava B, Smith C, Boughner JC, Rasskin-Gutman D (2015). Anatomical network comparison of human upper and lower, newborn and adult, and normal and abnormal limbs, with notes on development, pathology and limb serial homology vs. homoplasy. PLOS ONE.

[42] Esteve-Altava B, Marugán-Lobón J, Botella H, Bastir M, Rasskin-Gutman D (2013). Grist for Riedl’s mill: a network model perspective on the integration and modularity of the human skull. J. Exp. Zoolog. B Mol. Dev. Evol..

[43] Leche, W. *Über die Säugethiergattung Galeopithecus: eine morphologische untersuchung*. **11**, (PA Norstedt & Söner, 1886).

[44] Holmgren N (1952). An embryological analysis of the mammalian carpus and its bearing upon the question of the origin of the tetrapod limb. Acta Zool..

[45] George RM (1977). The limb musculature of the Tupaiidae. Primates.

[46] Stafford BJ, Thorington RW (1998). Carpal development and morphology in archontan mammals. J. Morphol..

[47] Kawashima T, Murakami K, Takayanagi M, Sato F (2012). Evolutionary transformation of the cervicobrachial plexus in the colugo (Cynocephalidae: Dermoptera) with a comparison to treeshrews (Tupaiidae: Scandentia) and strepsirrhines (Strepsirrhini: Primates). Folia Morphol..

[48] Chester SGB, Bloch JI, Boyer DM, Clemens WA (2015). Oldest known euarchontan tarsals and affinities of Paleocene *Purgatorius* to Primates. Proc. Natl. Acad. Sci..

[49] R Core Team. *R: A language and environment for statistical computing*. (R Foundation for Statistical Computing, 2016).

[50] Csardi G, Nepusz T (2006). The igraph software package for complex network research. InterJournal Complex Syst..

[51] Pons, P. & Latapy, M. Computing communities in large networks using random walks (long version). *arXiv:physics/0512106* (2005).

[52] Newman, M. E. J. & Girvan, M. Finding and evaluating community structure in networks. *Phys. Rev. E* **69** (2004). 10.1103/PhysRevE.69.026113 14995526

[53] Adkins RM, Walton AH, Honeycutt RL (2003). Higher-level systematics of rodents and divergence time estimates based on two congruent nuclear genes. Mol. Phylogenet. Evol..

[54] Esteve-Altava B, Boughner JC, Diogo R, Villmoare BA, Rasskin-Gutman D (2015). Anatomical network analysis shows decoupling of modular lability and complexity in the evolution of the primate skull. PLOS ONE.

[55] Revell L (2012). J. phytools: an R package for phylogenetic comparative biology (and other things). Methods Ecol. Evol..

[56] Felsenstein J (1985). Phylogenies and the comparative method. Am. Nat..

