# Supplementary material for: Comparison of musculoskeletal networks of the primate forelimb
Source: Sci Rep. 2017 Sep 5;7:10520. doi: 10.1038/s41598-017-09566-7 (PMC5585202; doi:10.1038/s41598-017-09566-7)
Supplement: Supplementary file 1 — Tables S1-S5 [file 41598_2017_9566_MOESM1_ESM.pdf]

Comparison of musculoskeletal networks of the primate forelimb

Julia Molnar*[1], Borja Esteve-Altava[1,2], Campbell Rolian[3], Rui Diogo[1]

1. Howard University College of Medicine, Numa Adams Building, 520 W Street NW, Washington, DC 20001
2. The Royal Veterinary College, Hawkshead Lane, Hatfield, Hertfordshire, AL97TA, UK
3. University of Calgary, 3330 Hospital Drive NW, Calgary, Alberta, Canada, T2N 4N1

Table S1. Skeletal specimens examined for this study. Institutional abbreviations: American Museum of Natural History (AMNH), Harvard University Museum of Comparative Zoology (UMCZ).

Institution	Genus	Species	Specimen	Sex
AMNH	*Saimiri*	*boliviensis boliviensis*	239872	M
AMNH	*Pithecia*	*pithecia*	149149	M
AMNH	*Callithrix*	*humeralifer*	77249	M
AMNH	*Tarsius*	*bancanus borneanus*	270	unknown
AMNH	*Aotus*	*trivirgatus vociferans*	239852	F
AMNH	*Lemur*	*catta*	48192 (NY Zoo)	F (juv)
AMNH	*Loris*	*lydekkerianus*	22718	M
AMNH	*Nycticebus*	*coucang*	212953	unknown
AMNH	*Propithecus*	*verreauxi*	170491	F
AMNH	*Macaca*	*nemestrina*	106563	M
AMNH	*Papio*	*hamadryas anubis*	80297	F
AMNH	*Cercopithecus*	*mitis*	52368	F
AMNH	*Hylobates*	*lar entelloides*	148207	F
AMNH	*Pongo*	*pygmaeus*	35549	F
AMNH	*Pan*	*troglodytes*	51381	M
AMNH	*Gorilla*	*gorilla*	90194	M
AMNH	*Procolobus*	*badius*	54279	unknown
UMCZ	*Cynocephalus*	*volans*	6261	unknown
UMCZ	*Cynocephalus*	*volans*	6262	unknown
UMCZ	*Cynocephalus*	*volans*	6263	unknown
UMCZ	*Cynocephalus*	*volans*	6264	unknown
UMCZ	*Tupaia*	*glis ferruginea*	6941	unknown
UMCZ	*Tupaia*	*glis olivacea*	57546	unknown

Table S2. Best partitions identified using the random walk-trap algorithm – bones only.

	# Modules	Q value	Expected error
Mus	10	0.5351937	0.0726629
Tupaia	10	0.5540176	0.0685789
Cynocephalus	9	0.5529301	0.0772326
Lemur	11	0.4989712	0.071227
Propithecus	9	0.4686385	0.073421
Loris	9	0.4732143	0.0739879
Nycticebus	9	0.4872449	0.0736957
Tarsius	11	0.509434	0.0714119
Callithrix	11	0.4975995	0.0713297
Saimiri	9	0.49911	0.0750353
Aotus	11	0.504806	0.0714105
Pithecia	11	0.4975995	0.0713297
Colobus	11	0.4975207	0.0705468
Cercopithecus	10	0.5035076	0.0697171
Papio	10	0.5035076	0.0697171
Macaca	10	0.5196109	0.0681343
Hylobates	12	0.4996	0.0737474
Pongo	12	0.5089546	0.0750526
Gorilla	9	0.5	0.0797776
Pan troglodytes	9	0.5	0.0797776
Pan paniscus	9	0.5	0.0797776
Homo	9	0.5	0.0797776

Table S3. Best partitions identified using the random walk-trap algorithm – muscles + bones.

	# Modules	Q value	Expected error
Mus	7	0.5572108	0.0311267
Tupaia	9	0.5408615	0.0307808
Cynocephalus	8	0.5237653	0.0294804
Lemur	7	0.5895846	0.0285508
Propithecus	7	0.5855408	0.0291634
Loris	11	0.5445332	0.0322082
Nycticebus	8	0.5635949	0.0298661
Tarsius	7	0.5850779	0.0272189
Callithrix	5	0.5612032	0.0285783
Saimiri	6	0.5717486	0.0289213
Aotus	5	0.579162	0.0279535
Pithecia	4	0.5665765	0.0259911
Colobus	8	0.5791219	0.0299456
Cercopithecus	11	0.5684007	0.0309378
Papio	11	0.5635484	0.0306035
Macaca	10	0.5733206	0.0295825
Hylobates	8	0.5582233	0.031073
Pongo	6	0.5255786	0.031316
Gorilla	8	0.5374631	0.0324379
Pan troglodytes	11	0.5391254	0.0317625
Pan paniscus	7	0.5510098	0.0316175
Homo	5	0.5795726	0.0297993

Table S4. Bone-bone connectivity modules identified in the forelimb. Colors correspond to text Figure 3.

ID	module	p-value	Elements
Aotus			
1	TRUE	0.07865	proximal.phalanx.5, middle.phalanx.5, distal.phalanx.5
2	TRUE	0.07865	metacarpal.1, proximal.phalanx.1, distal.phalanx.1
3	TRUE	0.07865	proximal.phalanx.4, middle.phalanx.4, distal.phalax.4
4	TRUE	0.07865	proximal.phalanx.2, middle.phalanx.2, distal.phalanx.2
5	TRUE	0.07865	proximal.phalanx.3, middle.phalanx.3, distal.phalanx.3
6	TRUE	0.07235	humerus, radius, ulna, triquetrum, pisiform
7	TRUE	0.00043	scaphoid, lunate, trapezium, accessory.on.trap, trapezoid, centrale, capitate, metacarpal.2, metacarpal.3
8	TRUE	1	ribs, sternum
9	TRUE	1	scapula, clavicle
10	TRUE	0.60187	hamate, metacarpal.4, metacarpal.5
11	TRUE	0.30854	occipital, vertebrae
Callithrix			
1	TRUE	0.00425	scaphoid, lunate, trapezium, accessory.on.trap, trapezoid, centrale, capitate, metacarpal.2
2	TRUE	0.07865	metacarpal.1, proximal.phalanx.1, distal.phalanx.1
3	TRUE	0.07865	proximal.phalanx.5, middle.phalanx.5, distal.phalanx.5
4	TRUE	0.07865	proximal.phalanx.4, middle.phalanx.4, distal.phalax.4
5	TRUE	0.07865	proximal.phalanx.2, middle.phalanx.2, distal.phalanx.2
6	TRUE	0.07865	proximal.phalanx.3, middle.phalanx.3, distal.phalanx.3

7	TRUE	0.02938	humerus, radius, ulna, triquetrum, pisiform
8	TRUE	1	ribs, sternum
9	TRUE	0.7294	hamate, metacarpal.3, metacarpal.4, metacarpal.5
10	TRUE	1	scapula, clavicle
11	TRUE	0.30854	occipital, vertebrae
Cercopithecus			
1	TRUE	0.04318	ribs, sternum, scapula, clavicle
2	TRUE	8.00E-05	scaphoid, trapezium, trapezoid, centrale, capitate, hamate, metacarpal.2, metacarpal.3, metacarpal.4, metacarpal.5
3	TRUE	0.07865	proximal.phalanx.5, middle.phalanx.5, distal.phalanx.5
4	TRUE	0.07865	proximal.phalanx.4, middle.phalanx.4, distal.phalax.4
5	TRUE	0.07865	proximal.phalanx.2, middle.phalanx.2, distal.phalanx.2
6	TRUE	0.07865	proximal.phalanx.3, middle.phalanx.3, distal.phalanx.3
7	TRUE	0.0068	humerus, radius, ulna, lunate, triquetrum, pisiform
8	TRUE	0.30854	proximal.phalanx.1, distal.phalanx.1
9	TRUE	0.30854	occipital, vertebrae
10	TRUE	0.93319	accessory.on.trap, metacarpal.1
Colobus			
1	TRUE	0.07865	metacarpal.1, proximal.phalanx.1, distal.phalanx.1
2	TRUE	0.07865	proximal.phalanx.5, middle.phalanx.5, distal.phalanx.5
3	TRUE	0.07865	proximal.phalanx.4, middle.phalanx.4, distal.phalax.4
4	TRUE	0.07865	proximal.phalanx.2, middle.phalanx.2, distal.phalanx.2
5	TRUE	0.07865	proximal.phalanx.3, middle.phalanx.3, distal.phalanx.3
6	TRUE	0.00073	scaphoid, lunate, trapezium, prepollex, trapezoid, centrale, capitate, metacarpal.2, metacarpal.3
7	TRUE	0.02938	humerus, radius, ulna, triquetrum, pisiform
8	TRUE	1	ribs, sternum
9	TRUE	1	scapula, clavicle
10	TRUE	0.82711	hamate, metacarpal.4, metacarpal.5
11	TRUE	0.30854	occipital, vertebrae
Cynocephalus			
1	TRUE	0.02353	metacarpal.5, proximal.phalanx.5, middle.phalanx.5, distal.phalanx.5
2	TRUE	0.02353	metacarpal.3, proximal.phalanx.3, middle.phalanx.3, distal.phalanx.3
3	TRUE	0.04318	ribs, sternum, scapula, clavicle
4	TRUE	0.001	scaphoid, lunate, trapezium, trapezoid, capitate, hamate, metacarpal.2, metacarpal.4
5	TRUE	0.07865	metacarpal.1, proximal.phalanx.1, distal.phalanx.1
6	TRUE	0.07865	proximal.phalanx.2, middle.phalanx.2, distal.phalanx.2
7	TRUE	0.07865	proximal.phalanx.4, middle.phalanx.4, distal.phalax.4
8	TRUE	0.02938	humerus, radius, ulna, triquetrum, pisiform
9	TRUE	0.30854	occipital, vertebrae

Gorilla

1	TRUE	3.00E-05	scaphoid, lunate, triquetrum, pisiform, trapezium, trapezoid, capitate, hamate, metacarpal.2, metacarpal.3, metacarpal.4, metacarpal.5
2	TRUE	0.04318	ribs, sternum, scapula, clavicle
3	TRUE	0.07865	proximal.phalanx.5, middle.phalanx.5, distal.phalanx.5
4	TRUE	0.07865	metacarpal.1, proximal.phalanx.1, distal.phalanx.1
5	TRUE	0.07865	proximal.phalanx.2, middle.phalanx.2, distal.phalanx.2
6	TRUE	0.07865	proximal.phalanx.4, middle.phalanx.4, distal.phalax.4
7	TRUE	0.07865	proximal.phalanx.3, middle.phalanx.3, distal.phalanx.3
8	TRUE	0.09835	humerus, radius, ulna
9	TRUE	0.30854	occipital, vertebrae

Homo

1	TRUE	3.00E-05	trapezoid, trapezium, scaphoid, lunate, triquetrum, pisiform, hamate, capitate, metacarpal.2, metacarpal.3, metacarpal.4, metacarpal.5
2	TRUE	0.04318	sternum, ribs, clavicle, scapula
3	TRUE	0.07865	proximal.phalanx.5, middle.phalanx.5, distal.phalanx.5
4	TRUE	0.07865	metacarpal.1, proximal.phalanx.1, distal.phalanx.1
5	TRUE	0.07865	proximal.phalanx.2, middle.phalanx.2, distal.phalanx.2
6	TRUE	0.07865	proximal.phalanx.4, middle.phalanx.4, distal.phalanx.4
7	TRUE	0.07865	proximal.phalanx.3, middle.phalanx.3, distal.phalanx.3
8	TRUE	0.09835	humerus, radius, ulna
9	TRUE	0.30854	occipital, vertebrae

Hylobates

1	TRUE	0.07865	proximal.phalanx.5, middle.phalanx.5, distal.phalanx.5
2	TRUE	0.07865	proximal.phalanx.4, middle.phalanx.4, distal.phalax.4
3	TRUE	0.07865	metacarpal.1, proximal.phalanx.1, distal.phalanx.1
4	TRUE	0.07865	proximal.phalanx.2, middle.phalanx.2, distal.phalanx.2
5	TRUE	0.07865	proximal.phalanx.3, middle.phalanx.3, distal.phalanx.3
6	TRUE	0.00067	scaphoid, lunate, trapezium, trapezoid, centrale, capitate, metacarpal.2, metacarpal.3
7	TRUE	1	ribs, sternum
8	TRUE	0.65845	triquetrum, pisiform
9	TRUE	1	scapula, clavicle
10	TRUE	0.60187	hamate, metacarpal.4, metacarpal.5
11	TRUE	0.09382	humerus, radius, ulna
12	TRUE	0.30854	occipital, vertebrae

Lemur

1	TRUE	0.07865	proximal.phalanx.5, middle.phalanx.5, distal.phalanx.5
2	TRUE	0.07865	metacarpal.1, proximal.phalanx.1, distal.phalanx.1
3	TRUE	0.07865	proximal.phalanx.4, middle.phalanx.4, distal.phalax.4
4	TRUE	0.07865	proximal.phalanx.2, middle.phalanx.2, distal.phalanx.2

5	TRUE	0.07865	proximal.phalanx.3, middle.phalanx.3, distal.phalanx.3
6	TRUE	0.02938	humerus, radius, ulna, triquetrum, pisiform
7	TRUE	1	ribs, sternum
8	TRUE	0.001	scaphoid, lunate, trapezium, trapezoid, centrale, capitate, metacarpal.2, metacarpal.3
9	TRUE	1	scapula, clavicle
10	TRUE	0.82711	hamate, metacarpal.4, metacarpal.5
11	TRUE	0.30854	occipital, vertebrae
Loris			
1	TRUE	0.04318	ribs, sternum, scapula, clavicle
2	TRUE	0.03366	humerus, radius, ulna, lunate, triquetrum, pisiform, hamate
3	TRUE	0.00125	scaphoid, trapezium, trapezoid, centrale, capitate, metacarpal.2, metacarpal.3, metacarpal.4, metacarpal.5
4	TRUE	0.07865	proximal.phalanx.5, middle.phalanx.5, distal.phalanx.5
5	TRUE	0.07865	proximal.phalanx.4, middle.phalanx.4, distal.phalax.4
6	TRUE	0.07865	metacarpal.1, proximal.phalanx.1, distal.phalanx.1
7	TRUE	0.07865	proximal.phalanx.2, middle.phalanx.2, distal.phalanx.2
8	TRUE	0.07865	proximal.phalanx.3, middle.phalanx.3, distal.phalanx.3
9	TRUE	0.30854	occipital, vertebrae
Macaca			
1	TRUE	0.04318	ribs, sternum, scapula, clavicle
2	TRUE	0.00086	scaphoid, trapezium, accessory.on.trap, trapezoid, centrale, capitate, metacarpal.1, metacarpal.2, metacarpal.3
3	TRUE	0.07865	proximal.phalanx.5, middle.phalanx.5, distal.phalanx.5
4	TRUE	0.07865	proximal.phalanx.4, middle.phalanx.4, distal.phalax.4
5	TRUE	0.07865	proximal.phalanx.2, middle.phalanx.2, distal.phalanx.2
6	TRUE	0.07865	proximal.phalanx.3, middle.phalanx.3, distal.phalanx.3
7	TRUE	0.01052	humerus, radius, ulna, lunate, triquetrum, pisiform
8	TRUE	0.60187	hamate, metacarpal.4, metacarpal.5
9	TRUE	0.30854	proximal.phalanx.1, distal.phalanx.1
10	TRUE	0.30854	occipital, vertebrae
Mus			
1	TRUE	0.04318	sternum, ribs, clavicle, scapula
2	TRUE	0.08062	humerus, radius, ulna, triquetrum, pisiform
3	TRUE	0.07865	proximal.phalanx.5, middle.phalanx.5, distal.phalanx.5
4	TRUE	0.07865	proximal.phalanx.4, middle.phalanx.4, distal.phalanx.4
5	TRUE	0.07865	proximal.phalanx.2, middle.phalanx.2, distal.phalanx.2
6	TRUE	0.07865	proximal.phalanx.3, middle.phalanx.3, distal.phalanx.3
7	TRUE	0.00689	trapezoid, trapezium, central.bone, scapholunate, falciform, capitate, metacarpal.1, metacarpal.2
8	TRUE	0.14247	hamate, metacarpal.3, metacarpal.4, metacarpal.5

9	TRUE	0.30854	proximal.phalanx.1, distal.phalanx.1
10	TRUE	0.30854	occipital, vertebrae

Nycticebus

1	TRUE	0.04318	ribs, sternum, scapula, clavicle
2	TRUE	0.07865	proximal.phalanx.5, middle.phalanx.5, distal.phalanx.5
3	TRUE	0.07865	proximal.phalanx.2, middle.phalanx.2, distal.phalanx.2
4	TRUE	0.07865	proximal.phalanx.4, middle.phalanx.4, distal.phalax.4
5	TRUE	0.07865	metacarpal.1, proximal.phalanx.1, distal.phalanx.1
6	TRUE	0.07865	proximal.phalanx.3, middle.phalanx.3, distal.phalanx.3
7	TRUE	3.00E-05	scaphoid, lunate, trapezium, trapezoid, centrale, capitate, hamate, metacarpal.2, metacarpal.3, metacarpal.4, metacarpal.5
8	TRUE	0.02938	humerus, radius, ulna, triquetrum, pisiform
9	TRUE	0.30854	occipital, vertebrae

Pan paniscus

1	TRUE	3.00E-05	scaphoid, lunate, triquetrum, pisiform, trapezium, trapezoid, capitate, hamate, metacarpal.2, metacarpal.3, metacarpal.4, metacarpal.5
2	TRUE	0.04318	ribs, sternum, scapula, clavicle
3	TRUE	0.07865	proximal.phalanx.5, middle.phalanx.5, distal.phalanx.5
4	TRUE	0.07865	metacarpal.1, proximal.phalanx.1, distal.phalanx.1
5	TRUE	0.07865	proximal.phalanx.2, middle.phalanx.2, distal.phalanx.2
6	TRUE	0.07865	proximal.phalanx.4, middle.phalanx.4, distal.phalax.4
7	TRUE	0.07865	proximal.phalanx.3, middle.phalanx.3, distal.phalanx.3
8	TRUE	0.09835	humerus, radius, ulna
9	TRUE	0.30854	occipital, vertebrae

Pan paniscus

1	TRUE	3.00E-05	scaphoid, lunate, triquetrum, pisiform, trapezium, trapezoid, capitate, hamate, metacarpal.2, metacarpal.3, metacarpal.4, metacarpal.5
2	TRUE	0.04318	ribs, sternum, scapula, clavicle
3	TRUE	0.07865	proximal.phalanx.5, middle.phalanx.5, distal.phalanx.5
4	TRUE	0.07865	metacarpal.1, proximal.phalanx.1, distal.phalanx.1
5	TRUE	0.07865	proximal.phalanx.2, middle.phalanx.2, distal.phalanx.2
6	TRUE	0.07865	proximal.phalanx.4, middle.phalanx.4, distal.phalax.4
7	TRUE	0.07865	proximal.phalanx.3, middle.phalanx.3, distal.phalanx.3
8	TRUE	0.09835	humerus, radius, ulna
9	TRUE	0.30854	occipital, vertebrae

Papio

1	TRUE	0.04318	ribs, sternum, scapula, clavicle
2	TRUE	8.00E-05	scaphoid, trapezium, trapezoid, centrale, capitate, hamate, metacarpal.2, metacarpal.3, metacarpal.4, metacarpal.5
3	TRUE	0.07865	proximal.phalanx.5, middle.phalanx.5, distal.phalanx.5
4	TRUE	0.07865	proximal.phalanx.4, middle.phalanx.4, distal.phalax.4

5	TRUE	0.07865	proximal.phalanx.2, middle.phalanx.2, distal.phalanx.2
6	TRUE	0.07865	proximal.phalanx.3, middle.phalanx.3, distal.phalanx.3
7	TRUE	0.0068	humerus, radius, ulna, lunate, triquetrum, pisiform
8	TRUE	0.30854	proximal.phalanx.1, distal.phalanx.1
9	TRUE	0.30854	occipital, vertebrae
10	TRUE	0.93319	accessory.on.trap, metacarpal.1
Pithecia			
1	TRUE	0.00425	scaphoid, lunate, trapezium, accessory.on.trap, trapezoid, centrale, capitate, metacarpal.2
2	TRUE	0.07865	metacarpal.1, proximal.phalanx.1, distal.phalanx.1
3	TRUE	0.07865	proximal.phalanx.5, middle.phalanx.5, distal.phalanx.5
4	TRUE	0.07865	proximal.phalanx.4, middle.phalanx.4, distal.phalax.4
5	TRUE	0.07865	proximal.phalanx.2, middle.phalanx.2, distal.phalanx.2
6	TRUE	0.07865	proximal.phalanx.3, middle.phalanx.3, distal.phalanx.3
7	TRUE	0.02938	humerus, radius, ulna, triquetrum, pisiform
8	TRUE	1	ribs, sternum
9	TRUE	0.7294	hamate, metacarpal.3, metacarpal.4, metacarpal.5
10	TRUE	1	scapula, clavicle
11	TRUE	0.30854	occipital, vertebrae
Pongo			
1	TRUE	0.07865	proximal.phalanx.5, middle.phalanx.5, distal.phalanx.5
2	TRUE	0.07865	proximal.phalanx.4, middle.phalanx.4, distal.phalax.4
3	TRUE	0.07865	metacarpal.1, proximal.phalanx.1, distal.phalanx.1
4	TRUE	0.07865	proximal.phalanx.2, middle.phalanx.2, distal.phalanx.2
5	TRUE	0.07865	proximal.phalanx.3, middle.phalanx.3, distal.phalanx.3
6	TRUE	0.00067	scaphoid, lunate, trapezium, trapezoid, centrale, capitate, metacarpal.2, metacarpal.3
7	TRUE	1	ribs, sternum
8	TRUE	1	scapula, clavicle
9	TRUE	0.65845	triquetrum, pisiform
10	TRUE	0.60187	hamate, metacarpal.4, metacarpal.5
11	TRUE	0.09835	humerus, radius, ulna
12	TRUE	0.30854	occipital, vertebrae
Propithecus			
1	TRUE	0.04318	ribs, sternum, scapula, clavicle
2	TRUE	2.00E-05	scaphoid, lunate, trapezium, accessory.on.trap, trapezoid, centrale, capitate, hamate, metacarpal.2, metacarpal.3, metacarpal.4, metacarpal.5
3	TRUE	0.07865	metacarpal.1, proximal.phalanx.1, distal.phalanx.1
4	TRUE	0.07865	proximal.phalanx.5, middle.phalanx.5, distal.phalanx.5
5	TRUE	0.07865	proximal.phalanx.4, middle.phalanx.4, distal.phalax.4
6	TRUE	0.07865	proximal.phalanx.2, middle.phalanx.2, distal.phalanx.2

7	TRUE	0.07865	proximal.phalanx.3, middle.phalanx.3, distal.phalanx.3
8	TRUE	0.02938	humerus, radius, ulna, triquetrum, pisiform
9	TRUE	0.30854	occipital, vertebrae
Saimiri			
1	TRUE	0.04318	ribs, sternum, scapula, clavicle
2	TRUE	3.00E-05	scaphoid, lunate, trapezium, centrale, trapezoid, capitate, hamate, metacarpal.2, metacarpal.3, metacarpal.4, metacarpal.5
3	TRUE	0.07865	proximal.phalanx.5, middle.phalanx.5, distal.phalanx.5
4	TRUE	0.07865	proximal.phalanx.4, middle.phalanx.4, distal.phalax.4
5	TRUE	0.07865	metacarpal.1, proximal.phalanx.1, distal.phalanx.1
6	TRUE	0.07865	proximal.phalanx.2, middle.phalanx.2, distal.phalanx.2
7	TRUE	0.07865	proximal.phalanx.3, middle.phalanx.3, distal.phalanx.3
8	TRUE	0.02938	humerus, radius, ulna, triquetrum, pisiform
9	TRUE	0.30854	occipital, vertebrae
Tarsius			
1	TRUE	0.07865	proximal.phalanx.5, middle.phalanx.5, distal.phalanx.5
2	TRUE	0.07865	metacarpal.1, proximal.phalanx.1, distal.phalanx.1
3	TRUE	0.07865	proximal.phalanx.4, middle.phalanx.4, distal.phalax.4
4	TRUE	0.07865	proximal.phalanx.2, middle.phalanx.2, distal.phalanx.2
5	TRUE	0.07865	proximal.phalanx.3, middle.phalanx.3, distal.phalanx.3
6	TRUE	0.19042	lunate, triquetrum, hamate, metacarpal.4, metacarpal.5
7	TRUE	0.08602	humerus, radius, ulna, pisiform
8	TRUE	1	ribs, sternum
9	TRUE	0.00208	scaphoid, trapezium, trapezoid, centrale, capitate, metacarpal.2, metacarpal.3
10	TRUE	1	scapula, clavicle
11	TRUE	0.30854	occipital, vertebrae
Tupaia			
1	TRUE	0.04318	ribs, sternum, scapula, clavicle
2	TRUE	0.07865	proximal.phalanx.5, middle.phalanx.5, distal.phalanx.5
3	TRUE	0.07865	proximal.phalanx.4, middle.phalanx.4, distal.phalax.4
4	TRUE	0.07865	proximal.phalanx.2, middle.phalanx.2, distal.phalanx.2
5	TRUE	0.07865	proximal.phalanx.3, middle.phalanx.3, distal.phalanx.3
6	TRUE	0.00754	humerus, radius, ulna, scapholunate, triquetrum, pisiform
7	TRUE	0.00137	trapezium, centrale, trapezoid, capitate, metacarpal.1, metacarpal.2, metacarpal.3
8	TRUE	0.25249	hamate, metacarpal.4, metacarpal.5
9	TRUE	0.30854	proximal.phalanx.1, distal.phalanx.1
10	TRUE	0.30854	occipital, vertebrae

Table S5. Muscle-bone connectivity modules identified in the forelimb. Colors correspond to text Figures 4-5.

ID	module	p-value	Elements
Aotus			
1	TRUE	0	scaphoid, lunate, trapezium, accessory.on.trap, trapezoid, centrale, capitate, metacarpal.1, proximal.phalanx.1, metacarpal.2, proximal.phalanx.2, metacarpal.3, proximal.phalanx.3, flexor.carpi.radialis, contrahens.digitorum.2, adductor.pollicis, interosseus.dorsalis.1, interosseus.dorsalis.2, interosseus.dorsalis.3, interosseus.palmaris.1, flexor.pollicis.brevis, opponens.pollicis, abductor.pollicis.brevis, extensor.carpi.radialis.longus, extensor.carpi.radialis.brevis
2	TRUE	0.00174	occipital, vertebrae, ribs, sternum, clavicle, serratus.anterior, rhomboideus.major, rhomboideus.occipitalis, levator.scapulae, levator.claviculae, subclavius, pectoralis.major
3	TRUE	0.00012	triquetrum, pisiform, hamate, metacarpal.4, proximal.phalanx.4, metacarpal.5, proximal.phalanx.5, flexor.carpi.ulnaris, contrahens.digitorum.4, contrahens.digitorum.5, interosseus.dorsalis.4, interosseus.palmaris.2, interosseus.palmaris.3, flexor.digiti.minimi.brevis, opponens.digiti.minimi, abductor.digiti.minimi, extensor.carpi.ulnaris
4	TRUE	0	scapula, humerus, radius, ulna, pectoralis.minor, panniculus.carnosus, infraspinatus, supraspinatus, deltoideus., teres.minor, subscapularis, teres.major, latissimus.dorsi, dorsoepitrochlearis, triceps.brachii, brachialis, biceps.brachii, coracobrachialis, pronator.quadratus, palmaris.longus, epitrochleoanconeus, pronator.teres, brachioradialis, supinator, anconeus, abductor.pollicis.longus
5	TRUE	0	distal.phalanx.1, middle.phalanx.2, distal.phalanx.2, middle.phalanx.3, distal.phalanx.3, middle.phalanx.4, distal.phalax.4, middle.phalanx.5, distal.phalanx.5, flexor.digitorum.profundus, flexor.digitorum.superficialis, lumbrical.1, lumbrical.2, lumbrical.3, lumbrical.4, extensor.digitorum, extensor.digiti.minimi, extensor.indicis, extensor.pollicis.longus
Callithrix			
1	TRUE	0	scaphoid, lunate, trapezium, accessory.on.trap, trapezoid, centrale, capitate, metacarpal.1, proximal.phalanx.1, metacarpal.2, proximal.phalanx.2, metacarpal.3, proximal.phalanx.3, flexor.carpi.radialis, contrahens.digitorum.2, adductor.pollicis, interosseus.dorsalis.1, interosseus.dorsalis.2, interosseus.dorsalis.3, interosseus.palmaris.1, flexor.pollicis.brevis, abductor.pollicis.brevis, extensor.carpi.radialis.longus, extensor.carpi.radialis.brevis, abductor.pollicis.longus
2	TRUE	0.00322	occipital, vertebrae, ribs, sternum, clavicle, serratus.anterior, rhomboideus.major, rhomboideus.minor, rhomboideus.occipitalis, levator.scapulae, levator.claviculae, subclavius, pectoralis.major
3	TRUE	0.00196	hamate, metacarpal.4, proximal.phalanx.4, metacarpal.5, proximal.phalanx.5, contrahens.digitorum.4, contrahens.digitorum.5, interosseus.dorsalis.4, interosseus.palmaris.2, interosseus.palmaris.3, flexor.digiti.minimi.brevis, opponens.digiti.minimi, extensor.carpi.ulnaris
4	TRUE	0	scapula, humerus, radius, ulna, triquetrum, pisiform, pectoralis.minor, panniculus.carnosus, infraspinatus, supraspinatus, deltoideus., teres.minor, subscapularis, teres.major, latissimus.dorsi, dorsoepitrochlearis, triceps.brachii, brachialis, biceps.brachii, coracobrachialis, pronator.quadratus, palmaris.longus, flexor.carpi.ulnaris, epitrochleoanconeus, pronator.teres, brachioradialis, supinator, anconeus
5	TRUE	0	distal.phalanx.1, middle.phalanx.2, distal.phalanx.2, middle.phalanx.3, distal.phalanx.3, middle.phalanx.4, distal.phalax.4, middle.phalanx.5, distal.phalanx.5, flexor.digitorum.profundus, flexor.digitorum.superficialis, lumbrical.1, lumbrical.2, lumbrical.3, lumbrical.4, abductor.digiti.minimi, extensor.digitorum, extensor.digiti.minimi, extensor.indicis, extensor.pollicis.longus
Cercopithecus			
1	TRUE	0.01493	hamate, metacarpal.4, proximal.phalanx.4, metacarpal.5, contrahens.digitorum.4, flexor.brevis.profundus.7, flexor.brevis.profundus.8, intermetacarpal.4, opponens.digiti.minimi,

			extensor.carpi.ulnaris
2	TRUE	1.00E-05	scapula, humerus, radius, ulna, rhomboideus.minor, panniculus.carnosus, infraspinatus, supraspinatus, deltoideus., teres.minor, subscapularis, teres.major, triceps.brachii, brachialis, biceps.brachii, coracobrachialis, pronator.quadratus, palmaris.longus, epitrochleoanconeus, pronator.teres, brachioradialis, supinator, anconeus, abductor.pollicis.longus
3	TRUE	0.02655	occipital, vertebrae, rhomboideus.major, rhomboideus.occipitalis, levator.scapulae, levator.claviculae, latissimus.dorsi, dorsoepitrochlearis
4	TRUE	0.05137	proximal.phalanx.1, distal.phalanx.1, adductor.pollicis, flexor.brevis.profundus.2, flexor.pollicis.brevis, abductor.pollicis.brevis, extensor.pollicis.longus
5	TRUE	0.09609	metacarpal.2, proximal.phalanx.2, flexor.carpi.radialis, contrahens.digitorum.2, flexor.brevis.profundus.3, flexor.brevis.profundus.4, intermetacarpal.1, extensor.carpi.radialis.longus
6	TRUE	0	middle.phalanx.2, distal.phalanx.2, middle.phalanx.3, distal.phalanx.3, middle.phalanx.4, distal.phalax.4, middle.phalanx.5, distal.phalanx.5, flexor.digitorum.profundus, flexor.digitorum.superficialis, lumbrical.1, lumbrical.2, lumbrical.3, lumbrical.4, extensor.digitorum, extensor.digiti.minimi, extensor.indicis
7	TRUE	0.06416	scaphoid, lunate, triquetrum, pisiform, trapezoid, centrale, capitate, flexor.carpi.ulnaris
8	TRUE	0.17233	proximal.phalanx.5, contrahens.digitorum.5, flexor.brevis.profundus.9, flexor.digiti.minimi.brevis, abductor.digiti.minimi
9	TRUE	0.01308	ribs, sternum, clavicle, serratus.anterior, subclavius, pectoralis.major, pectoralis.minor
10	TRUE	0.03178	metacarpal.3, proximal.phalanx.3, flexor.brevis.profundus.5, flexor.brevis.profundus.6, intermetacarpal.2, intermetacarpal.3, extensor.carpi.radialis.brevis
11	TRUE	0.44047	trapezium, accessory.on.trap, metacarpal.1, opponens.pollicis

Colobus

1	TRUE	0.00012	scaphoid, lunate, trapezium, prepollex, trapezoid, centrale, capitate, metacarpal.1, metacarpal.2, proximal.phalanx.2, flexor.carpi.radialis, contrahens.digitorum.2, flexor.brevis.profundus.3, flexor.brevis.profundus.4, intermetacarpal.1, opponens.pollicis, extensor.carpi.radialis.longus, abductor.pollicis.longus
2	TRUE	3.00E-04	occipital, vertebrae, ribs, sternum, clavicle, serratus.anterior, rhomboideus, rhomboideus.occipitalis, levator.scapulae, levator.claviculae, subclavius, pectoralis.major, pectoralis.minor, latissimus.dorsi, dorsoepitrochlearis
3	TRUE	0.01144	triquetrum, pisiform, hamate, metacarpal.5, proximal.phalanx.5, flexor.carpi.ulnaris, contrahens.digitorum.5, flexor.brevis.profundus.9, flexor.digiti.minimi.brevis, opponens.digiti.minimi, abductor.digiti.minimi, extensor.carpi.ulnaris
4	TRUE	0	distal.phalanx.1, middle.phalanx.2, distal.phalanx.2, middle.phalanx.3, distal.phalanx.3, middle.phalanx.4, distal.phalax.4, middle.phalanx.5, distal.phalanx.5, flexor.digitorum.profundus, flexor.digitorum.superficialis, lumbrical.1, lumbrical.2, lumbrical.3, lumbrical.4, extensor.digitorum, extensor.digiti.minimi, extensor.indicis, extensor.pollicis.longus
5	TRUE	3.00E-05	scapula, humerus, radius, ulna, panniculus.carnosus, infraspinatus, supraspinatus, deltoideus., teres.minor, subscapularis, teres.major, triceps.brachii, brachialis, biceps.brachii, coracobrachialis, pronator.quadratus, palmaris.longus, epitrochleoanconeus, pronator.teres, brachioradialis, supinator, anconeus
6	TRUE	0.06901	metacarpal.4, proximal.phalanx.4, contrahens.digitorum.4, flexor.brevis.profundus.7, flexor.brevis.profundus.8, intermetacarpal.4
7	TRUE	0.17645	proximal.phalanx.1, adductor.pollicis, flexor.pollicis.brevis, abductor.pollicis.brevis
8	TRUE	0.02747	metacarpal.3, proximal.phalanx.3, flexor.brevis.profundus.5, flexor.brevis.profundus.6, intermetacarpal.2, intermetacarpal.3, extensor.carpi.radialis.brevis

Cynocephalus

1	TRUE	0.00045	scaphoid, lunate, pisiform, trapezium, trapezoid, capitate, hamate, metacarpal.1, proximal.phalanx.1, distal.phalanx.1, flexor.carpi.radialis, adductor.pollicis, flexor.pollicis.brevis, abductor.pollicis.brevis, extensor.carpi.radialis.longus, extensor.pollicis.longus, abductor.pollicis.longus
2	TRUE	0.05755	metacarpal.3, proximal.phalanx.3, metacarpal.4, proximal.phalanx.4, interosseus.dorsalis.2, interosseus.dorsalis.3, interosseus.dorsalis.4, interosseus.palmaris.2, extensor.carpi.radialis.brevis
3	TRUE	1.00E-05	scapula, humerus, radius, ulna, panniculus.carnosus, infraspinatus, supraspinatus, deltoideus.scapularis, teres.minor, subscapularis, teres.major, latissimus.dorsi, triceps.brachii, dorsoepitrochlearis, brachialis, biceps.brachii, coracobrachialis, palmaris.longus, epitrochleoanconeus, pronator.teres, brachioradialis, supinator, extensor.carpi.ulnaris, anconeus
4	TRUE	0.08085	triquetrum, metacarpal.5, proximal.phalanx.5, flexor.carpi.ulnaris, flexor.brevis.digitorum.manus, contrahens.digitorum.5, interosseus.palmaris.3, flexor.digiti.minimi.brevis, abductor.digiti.minimi
5	TRUE	0.30362	occipital, vertebrae, rhomboideus, levator.scapulae, levator.claviculae
6	TRUE	0.2289	metacarpal.2, proximal.phalanx.2, contrahens.digitorum.2, interosseus.dorsalis.1, interosseus.palmaris.1
7	TRUE	0	middle.phalanx.2, distal.phalanx.2, middle.phalanx.3, distal.phalanx.3, middle.phalanx.4, distal.phalax.4, middle.phalanx.5, distal.phalanx.5, flexor.digitorum.profundus, flexor.digitorum.superficialis, lumbrical.1, lumbrical.2, lumbrical.3, lumbrical.4, lumbrical.5, lumbrical.6, lumbrical.7, extensor.digitorum, extensor.digiti.minimi, extensor.indicis
8	TRUE	0.00563	ribs, sternum, clavicle, serratus.anterior, subclavius, pectoralis.major, pectoralis.minor, deltoideus.acromialis.et.clavicularis

Gorilla

1	TRUE	0.00079	occipital, vertebrae, ribs, sternum, clavicle, serratus.anterior, rhomboideus, levator.scapulae, levator.claviculae, subclavius, pectoralis.major, pectoralis.minor, latissimus.dorsi, dorsoepitrochlearis
2	TRUE	0.00382	scaphoid, lunate, trapezoid, capitate, hamate, metacarpal.2, metacarpal.3, proximal.phalanx.3, middle.phalanx.3, distal.phalanx.3, flexor.carpi.radialis, lumbrical.2, interosseus.dorsalis.2, interosseus.dorsalis.3, extensor.carpi.radialis.longus, extensor.carpi.radialis.brevis, extensor.digitorum
3	TRUE	0.00836	metacarpal.5, proximal.phalanx.5, middle.phalanx.5, distal.phalanx.5, lumbrical.4, interosseus.palmaris.4, flexor.digiti.minimi.brevis, opponens.digiti.minimi, extensor.carpi.ulnaris, extensor.digiti.minimi
4	TRUE	0.00062	scapula, humerus, radius, ulna, infraspinatus, supraspinatus, deltoideus., teres.minor, subscapularis, teres.major, triceps.brachii, brachialis, biceps.brachii, coracobrachialis, pronator.quadratus, flexor.digitorum.profundus, flexor.digitorum.superficialis, palmaris.longus, pronator.teres, brachioradialis, supinator, anconeus
5	TRUE	0.00083	trapezium, metacarpal.1, proximal.phalanx.1, distal.phalanx.1, adductor.pollicis, flexor.brevis.profundus.2, flexor.pollicis.brevis, opponens.pollicis, abductor.pollicis.brevis, extensor.pollicis.longus, abductor.pollicis.longus
6	TRUE	0.67553	triquetrum, pisiform, flexor.carpi.ulnaris, abductor.digiti.minimi
7	TRUE	0.00988	metacarpal.4, proximal.phalanx.4, middle.phalanx.4, distal.phalax.4, lumbrical.3, interosseus.palmaris.2, interosseus.dorsalis.4

Homo

1	TRUE	0.00196	occipital, sternum, ribs, vertebrae, clavicle, subclavius, pectoralis.major, pectoralis.minor, serratus.anterior, rhomboid.minor, rhomboid.major
2	TRUE	1.00E-05	middle.phalanx.2, middle.phalanx.3, middle.phalanx.4, middle.phalanx.5, distal.phalanx.2, distal.phalanx.3, distal.phalanx.4, distal.phalanx.5, flexor.digitorum.superficialis, flexor.digitorum.profundus, extensor.digitorum, extensor.digiti.minimi, extensor.indicis, lumbrical.1, lumbrical.2, lumbrical.3, lumbrical.4

3	TRUE	0	trapezoid, trapezium, scaphoid, capitate, metacarpal.1, metacarpal.2, metacarpal.3, proximal.phalanx.1, proximal.phalanx.2, proximal.phalanx.3, distal.phalanx.1, flexor.carpi.radialis, flexor.pollicis.longus, extensor.carpi.radialis.longus, extensor.carpi.radialis.brevis, extensor.pollicis.longus, adductor.pollicis, adductor.pollicis.accessorius, flexor.brevis.profundus.2, abductor.pollicis.brevis, flexor.pollicis.brevis, opponens.pollicis, interosseus.dorsalis.1, interosseus.dorsalis.2, interosseus.dorsalis.3, interosseus.palmaris.1
4	TRUE	5.00E-05	lunate, triquetrum, pisiform, hamate, metacarpal.4, metacarpal.5, proximal.phalanx.4, proximal.phalanx.5, flexor.carpi.ulnaris, opponens.digiti.minimi, flexor.digiti.minimi.brevis, abductor.digiti.minimi, interosseus.dorsalis.4, interosseus.palmaris.2, interosseus.palmaris.3
5	TRUE	1.00E-05	scapula, humerus, radius, ulna, deltoid, supraspinatus, infraspinatus, teres.minor, teres.major, subscapularis, levator.scapulae, latissimus.dorsi, biceps.brachii, coracobrachialis, brachialis, triceps.brachii, anconeus, pronator.teres, palmaris.longus, pronator.quadratus, brachioradialis, extensor.carpi.ulnaris, supinator, abductor.pollicis.longus, extensor.pollicis.brevis

Hylobates

1	TRUE	0.00024	triquetrum, pisiform, hamate, metacarpal.5, proximal.phalanx.5, middle.phalanx.5, distal.phalanx.5, flexor.carpi.ulnaris, lumbrical.4, contrahens.digitorum.5, interosseus.palmaris.3, flexor.digiti.minimi.brevis, opponens.digiti.minimi, abductor.digiti.minimi
2	TRUE	0.00035	occipital, vertebrae, ribs, sternum, clavicle, serratus.anterior, rhomboideus, levator.scapulae, levator.claviculae, subclavius, pectoralis.major, pectoralis.minor, latissimus.dorsi, dorsoepitrochlearis
3	TRUE	8.00E-04	scapula, humerus, radius, ulna, infraspinatus, supraspinatus, deltoideus., teres.minor, subscapularis, teres.major, triceps.brachii, brachialis, biceps.brachii, coracobrachialis, pronator.quadratus, flexor.digitorum.profundus, flexor.digitorum.superficialis, palmaris.longus, pronator.teres, brachioradialis, supinator, extensor.carpi.ulnaris, extensor.digitorum, extensor.digiti.minimi, abductor.pollicis.longus
4	TRUE	0.00476	metacarpal.4, proximal.phalanx.4, middle.phalanx.4, distal.phalax.4, lumbrical.3, contrahens.digitorum.4, interosseus.palmaris.2, interosseus.dorsalis.4, interosseus.accessorius.4
5	TRUE	0.01416	scaphoid, lunate, trapezium, trapezoid, centrale, capitate, metacarpal.1, metacarpal.2, flexor.carpi.radialis, adductor.pollicis, opponens.pollicis, extensor.carpi.radialis.longus, extensor.carpi.radialis.brevis, extensor.pollicis.brevis
6	TRUE	0.02728	proximal.phalanx.1, distal.phalanx.1, flexor.pollicis.longus, flexor.brevis.profundus.2, flexor.pollicis.brevis, abductor.pollicis.brevis, extensor.pollicis.longus
7	TRUE	0.01893	metacarpal.3, proximal.phalanx.3, middle.phalanx.3, distal.phalanx.3, lumbrical.2, interosseus.dorsalis.2, interosseus.dorsalis.3, interosseus.accessorius.2, interosseus.accessorius.3, extensor.indicis
8	TRUE	0.00425	proximal.phalanx.2, middle.phalanx.2, distal.phalanx.2, lumbrical.1, contrahens.digitorum.2, interosseus.palmaris.1, interosseus.dorsalis.1, interosseus.accessorius.1

Lemur

1	TRUE	0	occipital, vertebrae, scapula, humerus, radius, ulna, rhomboideus.major, rhomboideus.occipitalis, levator.scapulae, levator.claviculae, panniculus.carnosus, infraspinatus, supraspinatus, deltoideus.scapularis, deltoideus.acromialis.et.clavicularis, teres.minor, subscapularis, teres.major, latissimus.dorsi, triceps.brachii, dorsoepitrochlearis, brachialis, biceps.brachii, coracobrachialis, pronator.quadratus, palmaris.longus, epitrochleoanconeus, pronator.teres, brachioradialis, supinator, extensor.carpi.ulnaris, anconeus
2	TRUE	0.00267	metacarpal.3, proximal.phalanx.3, metacarpal.4, proximal.phalanx.4, flexor.brevis.profundus.5, flexor.brevis.profundus.6, flexor.brevis.profundus.7, flexor.brevis.profundus.8, intermetacarpal.2, intermetacarpal.3, intermetacarpal.4, extensor.carpi.radialis.brevis
3	TRUE	0.00011	scaphoid, lunate, trapezium, trapezoid, centrale, capitate, metacarpal.1, metacarpal.2, proximal.phalanx.2, flexor.carpi.radialis, contrahens.digitorum.2, flexor.brevis.profundus.3, flexor.brevis.profundus.4, intermetacarpal.1, opponens.pollicis, extensor.carpi.radialis.longus, abductor.pollicis.longus

4	TRUE	0.05478	proximal.phalanx.1, distal.phalanx.1, adductor.pollicis, flexor.brevis.profundus.2, flexor.pollicis.brevis, abductor.pollicis.brevis, extensor.pollicis.longus
5	TRUE	0.01294	triquetrum, pisiform, hamate, metacarpal.5, proximal.phalanx.5, flexor.carpi.ulnaris, contrahens.digitorum.5, flexor.brevis.profundus.9, flexor.digiti.minimi.brevis, opponens.digiti.minimi, abductor.digiti.minimi
6	TRUE	0	middle.phalanx.2, distal.phalanx.2, middle.phalanx.3, distal.phalanx.3, middle.phalanx.4, distal.phalax.4, middle.phalanx.5, distal.phalanx.5, flexor.digitorum.profundus, flexor.digitorum.superficialis, lumbrical.2, lumbrical.3, lumbrical.4, lumbrical.5, extensor.digitorum, extensor.digiti.minimi, extensor.indicis
7	TRUE	0.00425	ribs, sternum, clavicle, serratus.anterior, subclavius, pectoralis.major, pectoralis.minor

Loris

1	TRUE	0.01141	proximal.phalanx.1, distal.phalanx.1, adductor.pollicis, flexor.brevis.profundus.2, flexor.pollicis.brevis, abductor.pollicis.brevis, extensor.pollicis.longus
2	TRUE	0.02655	occipital, vertebrae, rhomboideus.major, rhomboideus.occipitalis, levator.scapulae, levator.claviculae, latissimus.dorsi, dorsoepitrochlearis
3	TRUE	0.29318	pisiform, proximal.phalanx.5, contrahens.digitorum.5, flexor.brevis.profundus.9, abductor.digiti.minimi
4	TRUE	3.00E-05	scapula, humerus, radius, ulna, triquetrum, panniculus.carnosus, infraspinatus, supraspinatus, deltoideus., teres.minor, subscapularis, teres.major, triceps.brachii, brachialis, biceps.brachii, coracobrachialis, pronator.quadratus, palmaris.longus, flexor.carpi.ulnaris, pronator.teres, brachioradialis, supinator, extensor.carpi.ulnaris, anconeus, abductor.pollicis.longus
5	TRUE	0.01649	metacarpal.2, proximal.phalanx.2, middle.phalanx.2, lumbrical.1, contrahens.digitorum.2, flexor.brevis.profundus.3, flexor.brevis.profundus.4, intermetacarpal.1, interosseus.accessorius.2, extensor.carpi.radialis.longus
6	TRUE	0.00415	distal.phalanx.2, middle.phalanx.3, distal.phalanx.3, middle.phalanx.4, distal.phalax.4, flexor.digitorum.profundus, flexor.digitorum.superficialis, lumbrical.2, lumbrical.3, interosseus.accessorius.4, extensor.digitorum, extensor.indicis
7	TRUE	0.01451	scaphoid, lunate, trapezium, trapezoid, centrale, capitate, metacarpal.1, flexor.carpi.radialis, opponens.pollicis
8	TRUE	0.14534	hamate, metacarpal.5, middle.phalanx.5, distal.phalanx.5, lumbrical.4, interosseus.accessorius.5, flexor.digiti.minimi.brevis, opponens.digiti.minimi, extensor.digiti.minimi
9	TRUE	0.01308	ribs, sternum, clavicle, serratus.anterior, subclavius, pectoralis.major, pectoralis.minor
10	TRUE	0.05589	metacarpal.3, proximal.phalanx.3, flexor.brevis.profundus.5, flexor.brevis.profundus.6, intermetacarpal.2, interosseus.accessorius.3, extensor.carpi.radialis.brevis
11	TRUE	0.03892	metacarpal.4, proximal.phalanx.4, flexor.brevis.profundus.7, flexor.brevis.profundus.8, intermetacarpal.3, intermetacarpal.4

Macaca

1	TRUE	4.00E-05	scaphoid, lunate, trapezium, accessory.on.trap, trapezoid, centrale, capitate, metacarpal.1, proximal.phalanx.1, distal.phalanx.1, adductor.pollicis, flexor.brevis.profundus.2, flexor.pollicis.brevis, opponens.pollicis, abductor.pollicis.brevis, extensor.pollicis.longus
2	TRUE	0.22086	triquetrum, pisiform, hamate, metacarpal.5, flexor.carpi.ulnaris, opponens.digiti.minimi, abductor.digiti.minimi, extensor.carpi.ulnaris
3	TRUE	0	scapula, humerus, radius, ulna, rhomboideus.minor, pectoralis.minor, panniculus.carnosus, infraspinatus, supraspinatus, teres.minor, subscapularis, teres.major, latissimus.dorsi, dorsoepitrochlearis, triceps.brachii, brachialis, biceps.brachii, coracobrachialis, pronator.quadratus, palmaris.longus, epitrochleoanconeus, pronator.teres, brachioradialis, supinator, anconeus, abductor.pollicis.longus
4	TRUE	0.05085	metacarpal.2, proximal.phalanx.2, flexor.carpi.radialis, contrahens.digitorum.2, flexor.brevis.profundus.3, flexor.brevis.profundus.4, intermetacarpal.1,

			extensor.carpi.radialis.longus
5	TRUE	0	middle.phalanx.2, distal.phalanx.2, middle.phalanx.3, distal.phalanx.3, middle.phalanx.4, distal.phalax.4, middle.phalanx.5, distal.phalanx.5, flexor.digitorum.profundus, flexor.digitorum.superficialis, lumbrical.1, lumbrical.2, lumbrical.3, lumbrical.4, extensor.digitorum, extensor.digiti.minimi, extensor.indicis
6	TRUE	0.16978	occipital, vertebrae, rhomboideus.major, rhomboideus.occipitalis, levator.scapulae, levator.claviculae
7	TRUE	0.06901	metacarpal.4, proximal.phalanx.4, contrahens.digitorum.4, flexor.brevis.profundus.7, flexor.brevis.profundus.8, intermetacarpal.4
8	TRUE	0.26785	proximal.phalanx.5, contrahens.digitorum.5, flexor.brevis.profundus.9, flexor.digiti.minimi.brevis
9	TRUE	0.01401	ribs, sternum, clavicle, serratus.anterior, subclavius, pectoralis.major, deltoideus.
10	TRUE	0.02747	metacarpal.3, proximal.phalanx.3, flexor.brevis.profundus.5, flexor.brevis.profundus.6, intermetacarpal.2, intermetacarpal.3, extensor.carpi.radialis.brevis
Mus			
1	TRUE	3.00E-04	metacarpal.2, metacarpal.3, proximal.phalanx.2, proximal.phalanx.3, flexor.carpi.radialis, lumbrical.2, lumbrical.3, contrahens.digiti.2, flexor.brevis.profundus.3, flexor.brevis.profundus.4, flexor.brevis.profundus.5, flexor.brevis.profundus.6, intermetacarpal.1, intermetacarpal.2, intermetacarpal.3, extensor.carpi.radialis.longus, extensor.carpi.radialis.brevis
2	TRUE	0.00041	trapezoid, trapezium, central.bone, scapholunate, falciform, capitate, metacarpal.1, proximal.phalanx.1, distal.phalanx.1, adductor.pollicis, flexor.pollicis.brevis, flexor.brevis.profundus.2, abductor.pollicis.brevis, abductor.pollicis.longus
3	TRUE	0.00057	occipital, sternum, ribs, vertebrae, clavicle, serratus.anterior, rhomboideus.major, rhomboideus.minor, rhomboideus.occipitalis, levator.scapulae, levator.claviculae, subclavius, pectoralis.major, pectoralis.minor, panniculus.carnosus
4	TRUE	0.0112	triquetrum, pisiform, hamate, metacarpal.5, proximal.phalanx.5, middle.phalanx.5, lumbrical.5, contrahens.digiti.5, flexor.brevis.profundus.9, flexor.digiti.minimi.brevis, opponens.digiti.minimi, abductor.digiti.minimi
5	TRUE	0	scapula, humerus, radius, ulna, infraspinatus, supraspinatus, deltoideus.scapularis, deltoideus.acromialis.et.clavicularis, teres.minor, subscapularis, teres.major, latissimus.dorsi, triceps.brachii, dorsoepitrochlearis, brachialis, biceps.brachii, coracobrachialis, pronator.quadratus, flexor.carpi.ulnaris, epitrochleoanconeus, pronator.teres, palmaris.brevis, supinator, extensor.carpi.ulnaris, anconeus
6	TRUE	0.00021	middle.phalanx.2, middle.phalanx.3, middle.phalanx.4, distal.phalanx.2, distal.phalanx.3, distal.phalanx.4, distal.phalanx.5, flexor.digitorum.profundus, flexor.digitorum.superficialis, palmaris.longus, extensor.digitorum, extensor.digiti.minimi, extensor.indicis
7	TRUE	0.02903	metacarpal.4, proximal.phalanx.4, lumbrical.4, flexor.brevis.profundus.7, flexor.brevis.profundus.8, intermetacarpal.4
Nycticebus			
1	TRUE	6.00E-05	scaphoid, lunate, trapezium, trapezoid, centrale, capitate, hamate, metacarpal.1, metacarpal.2, proximal.phalanx.2, flexor.carpi.radialis, contrahens.digitorum.2, intercapitulares.1, flexor.brevis.profundus.3, flexor.brevis.profundus.4, intermetacarpal.1, interosseus.accessorius.2, opponens.pollicis, extensor.carpi.radialis.longus
2	TRUE	0.00041	middle.phalanx.2, distal.phalanx.2, metacarpal.3, proximal.phalanx.3, middle.phalanx.3, distal.phalanx.3, flexor.digitorum.profundus, flexor.digitorum.superficialis, lumbrical.2, lumbrical.3, intercapitulares.2, intercapitulares.3, flexor.brevis.profundus.5, flexor.brevis.profundus.6, intermetacarpal.2, interosseus.accessorius.3, extensor.carpi.radialis.brevis, extensor.digitorum, extensor.indicis
3	TRUE	0.0068	metacarpal.5, proximal.phalanx.5, middle.phalanx.5, distal.phalanx.5, lumbrical.5, contrahens.digitorum.5, intercapitulares.4, flexor.brevis.profundus.9, interosseus.accessorius.5, flexor.digiti.minimi.brevis, opponens.digiti.minimi, abductor.digiti.minimi, extensor.digiti.minimi
4	TRUE	0.00643	proximal.phalanx.1, distal.phalanx.1, adductor.pollicis, flexor.brevis.profundus.2,

			flexor.pollicis.brevis, abductor.pollicis.brevis, extensor.pollicis.longus
5	TRUE	0.02655	occipital, vertebrae, rhomboideus.major, rhomboideus.occipitalis, levator.scapulae, levator.claviculae, latissimus.dorsi, dorsoepitrochlearis
6	TRUE	0	scapula, humerus, radius, ulna, triquetrum, pisiform, panniculus.carnosus, infraspinatus, supraspinatus, deltoideus, teres.minor, subscapularis, teres.major, triceps.brachii, brachialis, biceps.brachii, coracobrachialis, pronator.quadratus, palmaris.longus, flexor.carpi.ulnaris, pronator.teres, brachioradialis, supinator, extensor.carpi.ulnaris, anconeus, abductor.pollicis.longus
7	TRUE	0.00298	metacarpal.4, proximal.phalanx.4, middle.phalanx.4, distal.phalax.4, lumbrical.4, flexor.brevis.profundus.7, flexor.brevis.profundus.8, intermetacarpal.3, intermetacarpal.4, interosseus.accessorius.4
8	TRUE	0.01308	ribs, sternum, clavicle, serratus.anterior, subclavius, pectoralis.major, pectoralis.minor
Pan paniscus			
1	TRUE	0.00026	occipital, vertebrae, ribs, sternum, clavicle, serratus.anterior, rhomboideus, levator.scapulae, levator.claviculae, subclavius, pectoralis.major, pectoralis.minor, latissimus.dorsi
2	TRUE	0.00017	scaphoid, lunate, trapezoid, capitate, metacarpal.2, metacarpal.3, proximal.phalanx.3, middle.phalanx.3, distal.phalanx.3, flexor.carpi.radialis, lumbrical.2, flexor.brevis.profundus.5, flexor.brevis.profundus.6, intermetacarpal.2, intermetacarpal.3, extensor.carpi.radialis.longus, extensor.carpi.radialis.brevis
3	TRUE	1.00E-04	triquetrum, pisiform, hamate, metacarpal.5, proximal.phalanx.5, middle.phalanx.5, distal.phalanx.5, flexor.carpi.ulnaris, lumbrical.4, contrahens.digitorum.5, flexor.brevis.profundus.9, flexor.digiti.minimi.brevis, opponens.digiti.minimi, abductor.digiti.minimi, extensor.digiti.minimi
4	TRUE	0.00063	scapula, humerus, radius, ulna, infraspinatus, supraspinatus, deltoideus., teres.minor, subscapularis, teres.major, dorsoepitrochlearis, triceps.brachii, brachialis, biceps.brachii, coracobrachialis, pronator.quadratus, flexor.digitorum.profundus, flexor.digitorum.superficialis, palmaris.longus, pronator.teres, brachioradialis, supinator, extensor.carpi.ulnaris, anconeus, extensor.digitorum
5	TRUE	0.00122	trapezium, metacarpal.1, proximal.phalanx.1, distal.phalanx.1, adductor.pollicis, flexor.brevis.profundus.2, flexor.pollicis.brevis, opponens.pollicis, abductor.pollicis.brevis, extensor.pollicis.longus, abductor.pollicis.longus
6	TRUE	0.00118	metacarpal.4, proximal.phalanx.4, middle.phalanx.4, distal.phalax.4, lumbrical.3, contrahens.digitorum.4, flexor.brevis.profundus.7, flexor.brevis.profundus.8, intermetacarpal.4
7	TRUE	0.00058	proximal.phalanx.2, middle.phalanx.2, distal.phalanx.2, lumbrical.1, flexor.brevis.profundus.3, flexor.brevis.profundus.4, intermetacarpal.1, extensor.indicis
Pan troglodytes			
1	TRUE	0.00106	trapezium, metacarpal.1, proximal.phalanx.1, distal.phalanx.1, adductor.pollicis, flexor.brevis.profundus.2, flexor.pollicis.brevis, opponens.pollicis, abductor.pollicis.brevis, extensor.pollicis.longus, abductor.pollicis.longus
2	TRUE	0.16804	occipital, vertebrae, rhomboideus, levator.scapulae, levator.claviculae, latissimus.dorsi, dorsoepitrochlearis
3	TRUE	0.02433	ribs, sternum, clavicle, serratus.anterior, subclavius, pectoralis.major, pectoralis.minor
4	TRUE	0.31959	triquetrum, pisiform, hamate, metacarpal.5, flexor.carpi.ulnaris, flexor.digiti.minimi.brevis, opponens.digiti.minimi
5	TRUE	0.04763	humerus, radius, ulna, triceps.brachii, brachialis, pronator.quadratus, flexor.digitorum.profundus, flexor.digitorum.superficialis, palmaris.longus, epitrochleoanconeus, pronator.teres, brachioradialis, supinator, extensor.carpi.ulnaris, anconeus, extensor.digitorum
6	TRUE	0.3255	scaphoid, lunate, trapezoid, capitate, metacarpal.2, metacarpal.3, flexor.carpi.radialis, extensor.carpi.radialis.longus, extensor.carpi.radialis.brevis
7	TRUE	0.70666	scapula, infraspinatus, supraspinatus, deltoideus., teres.minor, subscapularis, teres.major,

			biceps.brachii, coracobrachialis
8	TRUE	0.00118	metacarpal.4, proximal.phalanx.4, middle.phalanx.4, distal.phalax.4, lumbrical.3, contrahens.digitorum.4, flexor.brevis.profundus.7, flexor.brevis.profundus.8, intermetacarpal.4
9	TRUE	0.00074	proximal.phalanx.5, middle.phalanx.5, distal.phalanx.5, lumbrical.4, contrahens.digitorum.5, flexor.brevis.profundus.9, abductor.digiti.minimi, extensor.digiti.minimi
10	TRUE	0.00058	proximal.phalanx.2, middle.phalanx.2, distal.phalanx.2, lumbrical.1, flexor.brevis.profundus.3, flexor.brevis.profundus.4, intermetacarpal.1, extensor.indicis
11	TRUE	0.00032	proximal.phalanx.3, middle.phalanx.3, distal.phalanx.3, lumbrical.2, flexor.brevis.profundus.5, flexor.brevis.profundus.6, intermetacarpal.2, intermetacarpal.3
Papio			
1	TRUE	0	scapula, humerus, radius, ulna, rhomboideus.minor, panniculus.carnosus, infraspinatus, supraspinatus, deltoideus., teres.minor, subscapularis, teres.major, latissimus.dorsi, dorsoepitrochlearis, triceps.brachii, brachialis, biceps.brachii, coracobrachialis, pronator.quadratus, palmaris.longus, epitrochleoanconeus, pronator.teres, brachioradialis, supinator, anconeus, abductor.pollicis.longus
2	TRUE	0.04983	hamate, metacarpal.5, proximal.phalanx.5, contrahens.digitorum.5, flexor.brevis.profundus.9, flexor.digiti.minimi.brevis, opponens.digiti.minimi, abductor.digiti.minimi, extensor.carpi.ulnaris
3	TRUE	0.09609	metacarpal.2, proximal.phalanx.2, flexor.carpi.radialis, contrahens.digitorum.2, flexor.brevis.profundus.3, flexor.brevis.profundus.4, intermetacarpal.1, extensor.carpi.radialis.longus
4	TRUE	0	middle.phalanx.2, distal.phalanx.2, middle.phalanx.3, distal.phalanx.3, middle.phalanx.4, distal.phalax.4, middle.phalanx.5, distal.phalanx.5, flexor.digitorum.profundus, flexor.digitorum.superficialis, lumbrical.1, lumbrical.2, lumbrical.3, lumbrical.4, extensor.digitorum, extensor.digiti.minimi, extensor.indicis
5	TRUE	0.07827	proximal.phalanx.1, distal.phalanx.1, adductor.pollicis, flexor.brevis.profundus.2, flexor.pollicis.brevis, abductor.pollicis.brevis, extensor.pollicis.longus
6	TRUE	0.16978	occipital, vertebrae, rhomboideus.major, rhomboideus.occipitalis, levator.scapulae, levator.claviculae
7	TRUE	0.06901	metacarpal.4, proximal.phalanx.4, contrahens.digitorum.4, flexor.brevis.profundus.7, flexor.brevis.profundus.8, intermetacarpal.4
8	TRUE	0.10223	scaphoid, lunate, triquetrum, pisiform, trapezoid, centrale, capitate, flexor.carpi.ulnaris
9	TRUE	0.01731	ribs, sternum, clavicle, serratus.anterior, subclavius, pectoralis.major, pectoralis.minor
10	TRUE	0.03178	metacarpal.3, proximal.phalanx.3, flexor.brevis.profundus.5, flexor.brevis.profundus.6, intermetacarpal.2, intermetacarpal.3, extensor.carpi.radialis.brevis
11	TRUE	0.44047	trapezium, accessory.on.trap, metacarpal.1, opponens.pollicis
Pithecia			
1	TRUE	0	scaphoid, lunate, trapezium, accessory.on.trap, trapezoid, centrale, capitate, metacarpal.1, proximal.phalanx.1, metacarpal.2, proximal.phalanx.2, metacarpal.3, proximal.phalanx.3, flexor.carpi.radialis, contrahens.digitorum.2, adductor.pollicis, interosseus.dorsalis.1, interosseus.dorsalis.2, interosseus.dorsalis.3, interosseus.palmaris.1, flexor.pollicis.brevis, opponens.pollicis, abductor.pollicis.brevis, extensor.carpi.radialis.longus, extensor.carpi.radialis.brevis
2	TRUE	0	occipital, vertebrae, ribs, sternum, scapula, clavicle, humerus, radius, ulna, serratus.anterior, rhomboideus, rhomboideus.occipitalis, levator.scapulae, levator.claviculae, subclavius, pectoralis.major, pectoralis.minor, infraspinatus, supraspinatus, deltoideus., teres.minor, subscapularis, teres.major, latissimus.dorsi, dorsoepitrochlearis, triceps.brachii, brachialis, biceps.brachii, coracobrachialis, pronator.quadratus, palmaris.longus, epitrochleoanconeus, pronator.teres, brachioradialis, supinator, anconeus, abductor.pollicis.longus

3	TRUE	0.00042	triquetrum, pisiform, hamate, metacarpal.4, proximal.phalanx.4, metacarpal.5, proximal.phalanx.5, flexor.carpi.ulnaris, contrahens.digitorum.4, contrahens.digitorum.5, interosseus.dorsalis.4, interosseus.palmaris.2, interosseus.palmaris.3, flexor.digiti.minimi.brevis, opponens.digiti.minimi, abductor.digiti.minimi, extensor.carpi.ulnaris
4	TRUE	0	distal.phalanx.1, middle.phalanx.2, distal.phalanx.2, middle.phalanx.3, distal.phalanx.3, middle.phalanx.4, distal.phalax.4, middle.phalanx.5, distal.phalanx.5, flexor.digitorum.profundus, flexor.digitorum.superficialis, lumbrical.1, lumbrical.2, lumbrical.3, lumbrical.4, extensor.digitorum, extensor.digiti.minimi, extensor.indicis, extensor.pollicis.longus
Pongo			
1	TRUE	0	triquetrum, pisiform, hamate, metacarpal.4, proximal.phalanx.4, middle.phalanx.4, distal.phalax.4, metacarpal.5, proximal.phalanx.5, middle.phalanx.5, distal.phalanx.5, flexor.digitorum.profundus, lumbrical.3, lumbrical.4, interosseus.palmaris.2, interosseus.palmaris.3, interosseus.dorsalis.4, flexor.digiti.minimi.brevis, opponens.digiti.minimi, abductor.digiti.minimi, extensor.digitorum, extensor.digiti.minimi
2	TRUE	0.02011	occipital, vertebrae, scapula, rhomboideus, rhomboideus.occipitalis, levator.scapulae, infraspinatus, supraspinatus, deltoideus., teres.minor, subscapularis, teres.major, latissimus.dorsi, dorsoepitrochlearis, biceps.brachii, coracobrachialis
3	TRUE	0.00025	humerus, radius, ulna, scaphoid, lunate, trapezoid, centrale, capitate, metacarpal.2, metacarpal.3, triceps.brachii, brachialis, pronator.quadratus, flexor.digitorum.superficialis, palmaris.longus, flexor.carpi.ulnaris, flexor.carpi.radialis, pronator.teres, extensor.carpi.radialis.longus, extensor.carpi.radialis.brevis, brachioradialis, supinator, extensor.carpi.ulnaris, anconeus
4	TRUE	1.00E-05	proximal.phalanx.2, middle.phalanx.2, distal.phalanx.2, proximal.phalanx.3, middle.phalanx.3, distal.phalanx.3, lumbrical.1, lumbrical.2, interosseus.palmaris.1, interosseus.dorsalis.1, interosseus.dorsalis.2, interosseus.dorsalis.3, extensor.indicis
5	TRUE	0.00046	trapezium, metacarpal.1, proximal.phalanx.1, distal.phalanx.1, adductor.pollicis, flexor.brevis.profundus.2, flexor.pollicis.brevis, opponens.pollicis, abductor.pollicis.brevis, extensor.pollicis.longus, abductor.pollicis.longus
6	TRUE	0.01207	ribs, sternum, clavicle, serratus.anterior, levator.claviculae, subclavius, pectoralis.major, pectoralis.minor
Propithecus			
1	TRUE	0.00148	occipital, vertebrae, ribs, sternum, clavicle, serratus.anterior, rhomboideus.major, levator.scapulae, levator.claviculae, subclavius, pectoralis.major, pectoralis.minor
2	TRUE	0.00267	metacarpal.3, proximal.phalanx.3, metacarpal.4, proximal.phalanx.4, flexor.brevis.profundus.5, flexor.brevis.profundus.6, flexor.brevis.profundus.7, flexor.brevis.profundus.8, intermetacarpal.2, intermetacarpal.3, intermetacarpal.4, extensor.carpi.radialis.brevis
3	TRUE	0.00015	scaphoid, lunate, trapezium, accessory.on.trap, trapezoid, centrale, capitate, metacarpal.1, metacarpal.2, proximal.phalanx.2, flexor.carpi.radialis, contrahens.digitorum.2, flexor.brevis.profundus.3, flexor.brevis.profundus.4, intermetacarpal.1, opponens.pollicis, extensor.carpi.radialis.longus, abductor.pollicis.longus
4	TRUE	0.01546	triquetrum, pisiform, hamate, metacarpal.5, proximal.phalanx.5, flexor.carpi.ulnaris, contrahens.digitorum.5, flexor.brevis.profundus.9, flexor.digiti.minimi.brevis, opponens.digiti.minimi, abductor.digiti.minimi
5	TRUE	0.02739	proximal.phalanx.1, distal.phalanx.1, adductor.pollicis, flexor.brevis.profundus.2, flexor.pollicis.brevis, abductor.pollicis.brevis, extensor.pollicis.longus
6	TRUE	0	scapula, humerus, radius, ulna, panniculus.carnosus, infraspinatus, supraspinatus, deltoideus, teres.minor, subscapularis, teres.major, latissimus.dorsi, triceps.brachii, dorsoepitrochlearis, brachialis, biceps.brachii, coracobrachialis, pronator.quadratus, palmaris.longus, epitrochleoanconeus, pronator.teres, brachioradialis, supinator, extensor.carpi.ulnaris, anconeus
7	TRUE	0	middle.phalanx.2, distal.phalanx.2, middle.phalanx.3, distal.phalanx.3, middle.phalanx.4, distal.phalax.4, middle.phalanx.5, distal.phalanx.5, flexor.digitorum.profundus, flexor.digitorum.superficialis, lumbrical.2, lumbrical.3, lumbrical.4, lumbrical.5, extensor.digitorum,

			extensor.digiti.minimi, extensor.indicis
Saimiri			
1	TRUE	0.00196	hamate, metacarpal.4, proximal.phalanx.4, metacarpal.5, proximal.phalanx.5, contrahens.digitorum.4, contrahens.digitorum.5, interosseus.dorsalis.4, interosseus.palmaris.2, interosseus.palmaris.3, flexor.digiti.minimi.brevis, opponens.digiti.minimi, extensor.carpi.ulnaris
2	TRUE	1.00E-05	scaphoid, lunate, trapezium, centrale, trapezoid, capitate, metacarpal.1, metacarpal.2, proximal.phalanx.2, metacarpal.3, proximal.phalanx.3, flexor.carpi.radialis, contrahens.digitorum.2, interosseus.dorsalis.1, interosseus.dorsalis.2, interosseus.dorsalis.3, interosseus.palmaris.1, opponens.pollicis, extensor.carpi.radialis.longus, extensor.carpi.radialis.brevis
3	TRUE	0	occipital, vertebrae, ribs, sternum, scapula, clavicle, serratus.anterior, rhomboideus, rhomboideus.occipitalis, levator.scapulae, levator.claviculae, subclavius, pectoralis.major, pectoralis.minor, infraspinatus, supraspinatus, deltoideus., teres.minor, subscapularis, teres.major, latissimus.dorsi, dorsoepitrochlearis, coracobrachialis
4	TRUE	0.00028	humerus, radius, ulna, triquetrum, pisiform, panniculus.carnosus, triceps.brachii, brachialis, biceps.brachii, pronator.quadratus, palmaris.longus, flexor.carpi.ulnaris, epitrochleoanconeus, pronator.teres, abductor.digiti.minimi, brachioradialis, supinator, anconeus, abductor.pollicis.longus
5	TRUE	0	distal.phalanx.1, middle.phalanx.2, distal.phalanx.2, middle.phalanx.3, distal.phalanx.3, middle.phalanx.4, distal.phalax.4, middle.phalanx.5, distal.phalanx.5, flexor.digitorum.profundus, flexor.digitorum.superficialis, lumbrical.1, lumbrical.2, lumbrical.3, lumbrical.4, extensor.digitorum, extensor.digiti.minimi, extensor.indicis, extensor.pollicis.longus
6	TRUE	0.30992	proximal.phalanx.1, adductor.pollicis, flexor.pollicis.brevis, abductor.pollicis.brevis
Tarsius			
1	TRUE	8.00E-05	triquetrum, pisiform, hamate, metacarpal.4, proximal.phalanx.4, metacarpal.5, proximal.phalanx.5, flexor.carpi.ulnaris, contrahens.digitorum.4, contrahens.digitorum.5, flexor.brevis.profundus.7, flexor.brevis.profundus.8, flexor.brevis.profundus.9, intermetacarpal.4, flexor.digiti.minimi.brevis, opponens.digiti.minimi, abductor.digiti.minimi
2	TRUE	0.00102	occipital, vertebrae, ribs, sternum, clavicle, serratus.anterior, rhomboideus.major, rhomboideus.occipitalis, levator.scapulae, levator.claviculae, subclavius, pectoralis.major, pectoralis.minor, latissimus.dorsi
3	TRUE	3.00E-05	scaphoid, lunate, trapezium, trapezoid, centrale, capitate, metacarpal.2, proximal.phalanx.2, metacarpal.3, proximal.phalanx.3, flexor.carpi.radialis, contrahens.digitorum.2, contrahens.digitorum.3, flexor.brevis.profundus.3, flexor.brevis.profundus.4, flexor.brevis.profundus.5, flexor.brevis.profundus.6, intermetacarpal.1, intermetacarpal.2, intermetacarpal.3, extensor.carpi.radialis.longus, extensor.carpi.radialis.brevis
4	TRUE	0	middle.phalanx.2, distal.phalanx.2, middle.phalanx.3, distal.phalanx.3, middle.phalanx.4, distal.phalax.4, middle.phalanx.5, distal.phalanx.5, flexor.digitorum.profundus, flexor.digitorum.superficialis, lumbrical.1, lumbrical.2, lumbrical.3, lumbrical.4, contrahens.digitorum.6, contrahens.digitorum.7, contrahens.digitorum.8, contrahens.digitorum.9, extensor.digitorum, extensor.digiti.minimi, extensor.indicis
5	TRUE	0	scapula, humerus, radius, ulna, panniculus.carnosus, infraspinatus, supraspinatus, deltoideus.scapularis., deltoideus.acromialis.et.clavicularis, teres.minor, subscapularis, teres.major, triceps.brachii, dorsoepitrochlearis, brachialis, biceps.brachii, coracobrachialis, pronator.quadratus, palmaris.longus, epitrochleanconeus, pronator.teres, brachioradialis, supinator, extensor.carpi.ulnaris, anconeus, abductor.pollicis.longus
6	TRUE	0.02051	metacarpal.1, proximal.phalanx.1, distal.phalanx.1, adductor.pollicis, flexor.brevis.profundus.2, flexor.pollicis.brevis, opponens.pollicis, abductor.pollicis.brevis, extensor.pollicis.longus
Tupaia			
1	TRUE	0	scapholunate, triquetrum, pisiform, trapezium, centrale, trapezoid, capitate, hamate, metacarpal.1, proximal.phalanx.1, distal.phalanx.1, metacarpal.2, metacarpal.3, metacarpal.4, flexor.carpi.ulnaris, flexor.carpi.radialis, flexor.brevis.digitorum.manus, adductor.pollicis, flexor.pollicis.brevis, abductor.pollicis.brevis, extensor.carpi.radialis.brevis, extensor.pollicis.longus, abductor.pollicis.longus

2	TRUE	0	middle.phalanx.2, distal.phalanx.2, middle.phalanx.3, distal.phalanx.3, middle.phalanx.4, distal.phalax.4, middle.phalanx.5, distal.phalanx.5, flexor.digitorum.profundus, flexor.digitorum.superficialis, lumbrical.1, lumbrical.2, lumbrical.3, lumbrical.4, extensor.digitorum, extensor.digiti.minimi, extensor.indicis
3	TRUE	0.16677	occipital, vertebrae, rhomboideus.major, rhomboideus.minor, rhomboideus.occipitalis, levator.scapulae, levator.claviculae, Atlantoscapularis.posticus.
4	TRUE	0	scapula, humerus, radius, ulna, panniculus.carnosus, infraspinatus, supraspinatus, deltoideus.scapularis, deltoideus.acromialis.et.clavicularis, teres.minor, subscapularis, teres.major, latissimus.dorsi, triceps.brachii, dorsoepitrochlearis, brachialis, biceps.brachii, coracobrachialis, pronator.quadratus, palmaris.longus, epitrochleoanconeus, pronator.teres, extensor.carpi.radialis.longus, brachioradialis, supinator, extensor.carpi.ulnaris, anconeus
5	TRUE	0.12345	metacarpal.5, proximal.phalanx.5, contrahens.digitorum.5, interosseus.palmaris.3, flexor.digiti.minimi.brevis, abductor.digiti.minimi
6	TRUE	0.01139	ribs, sternum, clavicle, serratus.anterior, subclavius, pectoralis.major, pectoralis.minor
7	TRUE	0.21784	proximal.phalanx.2, contrahens.digitorum.2, interosseus.dorsalis.1, interosseus.palmaris.1
8	TRUE	0.32655	proximal.phalanx.3, interosseus.dorsalis.2, interosseus.dorsalis.3
9	TRUE	0.32655	proximal.phalanx.4, interosseus.dorsalis.4, interosseus.palmaris.2
